# Revealing potential functions of hypothetical proteins induced by genistein in the symbiosis island of *Bradyrhizobium japonicum* commercial strain SEMIA 5079 (= CPAC 15)

**DOI:** 10.1186/s12866-022-02527-9

**Published:** 2022-05-05

**Authors:** Everton Geraldo Capote Ferreira, Douglas Fabiano Gomes, Caroline Vanzzo Delai, Marco Antônio Bacellar Barreiros, Luciana Grange, Elisete Pains Rodrigues, Liliane Marcia Mertz Henning, Fernando Gomes Barcellos, Mariangela Hungria

**Affiliations:** 1 Londrina State University (UEL), Celso Garcia Cid Road (PR 445), km 380, CEP 86057-970 Londrina, PR Brazil; 2 Embrapa Soja, Rodovia Carlos João Strass, C.P. 231, CEP 86001-970 Londrina, PR Brazil; 3 Total Biotecnologia, Rua Emílio Romani, 1190, CEP 81460-020 Curitiba, PR Brazil; 4 Federal University of Paraná (UFPR), Estrada dos Pioneiros 2153, CEP 85950-000 Palotina, PR Brazil

**Keywords:** Biological nitrogen fixation, Symbiosis, Functional inference, Gene expression

## Abstract

**Background:**

*Bradyrhizobium japonicum* strain SEMIA 5079 (= CPAC 15) is a nitrogen-fixing symbiont of soybean broadly used in commercial inoculants in Brazil. Its genome has about 50% of hypothetical (HP) protein-coding genes, many in the symbiosis island, raising questions about their putative role on the biological nitrogen fixation (BNF) process. This study aimed to infer functional roles to 15 HP genes localized in the symbiosis island of SEMIA 5079, and to analyze their expression in the presence of a *nod*-gene inducer.

**Results:**

A workflow of bioinformatics tools/databases was established and allowed the functional annotation of the HP genes. Most were enzymes, including transferases in the biosynthetic pathways of cobalamin, amino acids and secondary metabolites that may help in saprophytic ability and stress tolerance, and hydrolases, that may be important for competitiveness, plant infection, and stress tolerance. Putative roles for other enzymes and transporters identified are discussed. Some HP proteins were specific to the genus *Bradyrhizobium*, others to specific host legumes, and the analysis of orthologues helped to predict roles in BNF.

**Conclusions:**

All 15 HP genes were induced by genistein and high induction was confirmed in five of them, suggesting major roles in the BNF process.

**Supplementary information:**

The online version contains supplementary material available at 10.1186/s12866-022-02527-9.

## Background

Soybean [*Glycine max* (L.) Merrill] is one of the most important agricultural commodities worldwide due to its use in animal and human food and oil and biofuel production. Currently, the legume is the major crop in Brazil, with over 135 million tons of grains harvested in the 2020/2021 growing season [1]. Soybean grains contain about 40% of protein, thus requiring high amounts of nitrogen (N) to reach high yields. In the country, biological nitrogen fixation (BNF) achieved by inoculation of soybean seeds with *Bradyrhizobium* elite strains selected by the research over the past decades provides highs rates of fixed atmospheric nitrogen (N_2_), supplying the entire N needs from the plants [2]. Estimates are that approximately 12 Tg of mineral N (fertilizer) per year are saved in Brazil due to the BNF contribution to the soybean crop, representing an economy of about US$ 13 billion annually to the country [3].

Strain SEMIA 5079 (= CPAC 15) was isolated in 1992 from a Cerrado soil that originally had no compatible soybean rhizobia and was inoculated with strain SEMIA 566; as both strains share identical rep-PCR profiles and differ from all other strains from the germplasm bank, SEMIA 5079 is considered a natural variant of SEMIA 566 [2]. SEMIA 566 was isolated from plants inoculated with a North American inoculant in the late 1960s [4], and belongs to the same serogroup as USDA 123, highly competitive in the United States [2, 4]. The variant strain SEMIA 5079 shows higher N_2_ fixation than its parental, and since 1992 it composes the group of four *Bradyrhizobium* strains used in Brazilian inoculants [5]. Nowadays, SEMIA 5079 is the main strain composing more than 70 million doses of commercial inoculants sold per year in Brazil, used in more than 30 million hectares.

The genome of SEMIA 5079 was estimated at 9.58 Mbp, and is composed of one circular chromosome with two ribosomal operons and G + C content of 63.54%. The annotation predicted 4,203 putative genes with assigned function (48.6%) and 4,445 hypothetical CDSs (coding DNA sequences), totaling 8,648 predicted genes [6]. The most representative gene category is of amino acid metabolism (15.95%) and xenobiotic biodegradation and metabolism (13.24%). Other classes of genes associated with nutrient transport, iron acquisition, and indole acetic acid (IAA) metabolism potentially correlate with the higher saprophytic capacity and competitiveness properties of strain SEMIA 5079. The symbiosis island size was estimated at 700,212 bp, with 569 predicted CDSs, including all nodulation (*nod*, *noe*) and fixation (*fix* and *nif*) genes, 247 hypothetical ORFS, and 90 mobile elements. Additionally, the symbiosis island encompasses a probable non-functional hydrogenase operon, cytochromes for energy supply, ABC-transporters, and operons for secretion systems.

The *Bradyrhizobium*-soybean symbiosis is highly specific, with exchange of molecular signals between the plant and its symbiotic rhizobium. In the symbiosis, isoflavonoids (mainly genistein and daidzein) secreted by the soybean seeds and roots attract the specific *Bradyrhizobium* and activate the NodD family of transcription activators [7, 8]. The *nodD* gene activates the expression of common and host-specific *nod* genes. Following, rhizobia synthesize and secrete Nod factors (lipo-chitooligosaccharides, LCOs), which are signal molecules that interact with the soybean plant, inducing signal transduction cascades [9]. The recognition of Nod factors by kinase-like receptors on the legume root epidermal cells triggers the root hair deformation, resulting in nodule formation [10]. Besides being the primary inducers of *nod* gene expression, isoflavonoids are also related to chemotactic signals [11], induction of type III secretion system (T3SS) effectors [12], with the rapid proliferation of rhizobia [13], promotion of bacterial growth [14], and activation of rhizobial quorum sensing systems [15]. Altogether, these phenotypes may affect rhizobial competitiveness and nodulation [16, 17].

Nowadays, more than 187 genomes of *Bradyrhizobium* strains are publicly available, including several *B. japonicum* strains [18]. There has been a significant increase of new species identified in recent years, e.g. nine new *Bradyrhizobium* species have been described and their genomes released in the last two years [19]. Despite the increase of *Bradyrhizobium* genomes, there is still a lack of transcriptional studies supporting gene expression levels, their molecular functions during symbiosis, and their interactions with isoflavonoids.

The majority of the transcriptome studies has used strain USDA 110 (previously classified as *B. japonicum*, and later reclassified as the type strain of *B. diazoefficien*) as a model in micro and macro-arrays studies to assess the whole-genome expression profiling of soybean bradyrhizobia, evaluating gene expression levels in both bacteroids and free-living conditions [20–22]. Studies have also evaluated the response to drought stress [23] and gene expression when induced by genistein [24, 25], soybean seed extracts [24], soybean root exudates [16], coumestrol (a polyphenolic compound isolated from soybean roots) [26], and peribacteroid (PBS) solution purified from soybean root nodules [27].

Quantitative reverse transcription PCR (qRT-PCR) has been broadly chosen to evaluate the expression of target genes in rhizobia. Previous studies reported effects of isoflavonoids in the expression of several genes related to BNF (e.g. *nod* genes), T3SS effectors, efflux pump system in *B. diazoefficiens* USDA 110 [28, 29], and hypothetical protein-coding genes in *B. diazoefficiens* strain CPAC 7 (= SEMIA 5080), also used in commercial inoculants in Brazil [30]. Induction of the expression of *nodC*, *nodW*, and *nodP* genes in *B. japonicum* SEMIA 5079 [31], *nodD1*, *nodD2*, and *nodA* genes in *Bradyrhizobium liaoningense* CCBAU 05525 [32], and T3SS effectors expression in *Bradyrhizobium elkanii* strains [33] have also been shown.

Unfortunately, there is only one study reporting gene expression patterns in *B. japonicum* SEMIA 5079 [31]. Furthermore, the high number of genes located in the symbiosis island coding for hypothetical proteins points out to the need of conducting more transcriptional studies to understand the roles of these genes in the BNF process. As SEMIA 5079 is the most competitive strain used in commercial inoculants in Brazil [4], and as it belongs to the same serogroup as the very competitive USDA 123 [2], these studies can enlighten properties related to both the efficiency of BNF and competitiveness.

This study aimed to investigate gene expression levels of hypothetical protein-coding genes of the symbiosis island of *B. japonicum* strain SEMIA 5079 in response to genistein. We confirmed gene expression of different functional classes of genes, including enzymes, transporters, and potential effectors in response to genistein. We also provided a framework of helpful bioinformatics tools for functional annotation and curation of hypothetical proteins of rhizobia.

## Results

### Selection of hypothetical proteins coding genes and characterization of gene neighborhood

**Fig. 1:**
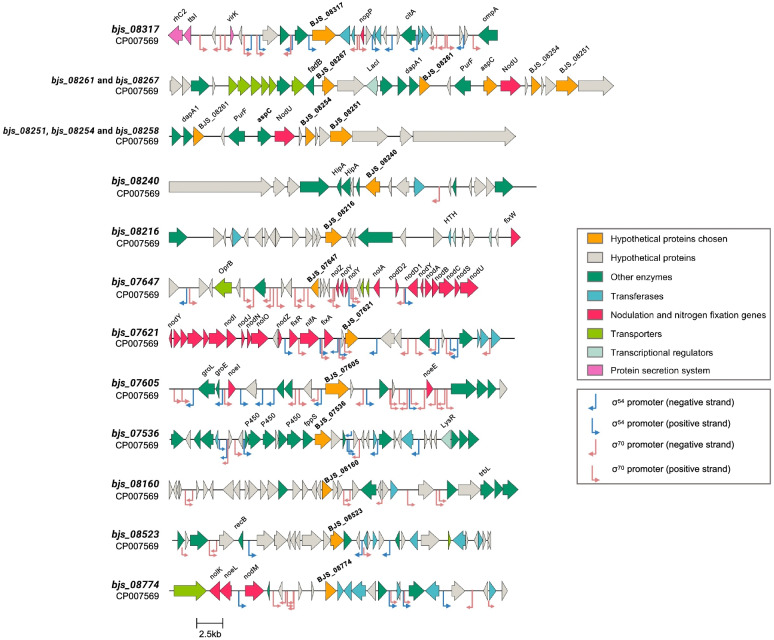
Genomic neighborhood of the 14 hypothetical protein-coding genes and of the *aspC* gene selected for this study in the symbiosis island of *Bradyrhizobium japonicum* strain SEMIA 5079 (= CPAC 15)

To define hypothetical protein-coding (HP) genes in the symbiosis island of *B. japonicum* SEMIA 5079 with possible roles in BNF and competitiveness, we searched for putative genes located relatively close to genes related to saprophytic capacity and/or BNF traits, such as the nodulation genes and secretion systems, located in a size range of 600-2,500 bp. By adopting this strategy, 15 HP genes were selected, but one of them was later identified as being *aspC*; information about the 15 HPs is shown in Table 1, while their genomic neighborhoods are shown in Fig. 1.

**Table 1:** Hypothetical proteins of the symbiosis island of *Bradyrhizobium japonicum* strain SEMIA 5079 (= CPAC 15) annotated in this study

Protein ID	Protein	Subcellular localization	SOSUI^a^	Signal Pep^b^	Molecular Function	Biological Process
**BJS_07536**	Squalene-hopene cyclase	Cytoplasmic	S	NSP	cyclizes squalene into hopanoid products	hopanoid (triterpenoid) metabolism
**BJS_07605**	5-methyltetrahydropteroyltriglutamate-homocysteine methyltransferase	Cytoplasmic	S	NSP	methyltransferase; zinc ion binding	methionine biosynthesis
**BJS_07621**	Glutathione-dependent formaldehyde-activating (GFA) enzyme	Cytoplasmic	S	NSP	carbon-sulfur lyase activity	metabolic process
**BJS_07647**	Porin protein	Outer Membrane	1 TMH	SP	non-specific channels for small hydrophilic molecules	molecules transport
**BJS_08160**	Non-homologous end joining protein Ku	Cytoplasmic	S	NSP	DNA binding	DNA recombination
**BJS_08216**	Hydrogenase maturation factor HypA	Cytoplasmic	S	NSP	nickel cation binding	cellular protein modification process
**BJS_08240**	Peptidase U49	Cytoplasmic	1 TMH	NSP	peptidase cleaves domain I of the elongation factor	catalytic mechanism
**BJS_08251**	Trypsin-like peptidase	Cytoplasmic	S	NSP	serine-type endopeptidase activity	proteolysis
**BJS_08254**	Trypsin-like peptidase	Extracellular	S	SP	serine-type endopeptidase activity	proteolysis
**BJS_08258** **(** ***aspC*** **)**	Aminotransferase class I/II-fold pyridoxal phosphate-dependent enzyme	Cytoplasmic	S	NSP	pyridoxal phosphate binding	biosynthetic process
**BJS_08261**	Succinate-semialdehyde dehydrogenase	Cytoplasmic	S	NSP	succinate-semialdehyde dehydrogenase	oxidation-reduction process
**BJS_08267**	Major Facilitator transporter	Inner Membrane	11 TMH	NSP	transmembrane transporter activity	molecules transport
**BJS_08317**	HxlR-type HTH transcription regulator	Cytoplasmic	S	NSP	transcription regulator	amino acid biosynthesis
**BJS_08523**	7-cyano-7-deazaguanine synthase	Cytoplasmic	1 TMH	NSP	ATP-dependent conversion of 7-carboxy-7-deazaguanine to 7-cyano-7-deazaguanine	queuosine biosynthetic process
**BJS_08774**	Acetylcholinesterase	Cytoplasmic	1 TMH	NSP	acetylcholinesterase tetramerization	glycerophospholipid metabolism

^a^ Prediction of soluble protein (S) or transmembrane helices structures (TMH); ^b^ Prediction of peptide signal, secreted protein (SP) or no-secreted protein (NSP)

The closest gene to the *bjs_07605* is the nodulation *noeI*, with a distance of 8,615 bp, followed by *noeE* (9,683 bp). The *bjs_07621* is located close to several BNF genes, the closest being *fixA*. The *bjs_07647* is located at 1,698 bp from *nolZ* gene, while *bjs_08317* is located at 2,361 bp from the *nopP* gene (type III secreted effector). Following, *bjs_08160* has as nearest gene the conjugal transfer gene *trbL* (15,779 bp), and *bjs_08216* is closer to *fixW* gene (16,189 bp). The *bjs_08251* and *bjs_08254* are located near to each other (1,469 bp), and are close to the 6-O-carbamoyl transferase (*nodU* gene), with a distance of 3,381 bp and 1,022 bp, respectively. We also selected the *aspC* gene (*bjs_08258*) in order to compare the expression of an annotated gene in our set of HP, and the gene is close to *nodU* (315 bp). Close to the *bjs_08267*, an enoyl-CoA hydratase (*fadB* gene, 810 bp) and a LacI family transcription regulator coding (*lacI*) were identified. Several ABC transporters spanning a ~ 5 kb region are also located close to *bjs_08267* (Fig. 1).

### Distribution of HPs between strains of *Bradyrhizobium* genus

**Fig. 2:**
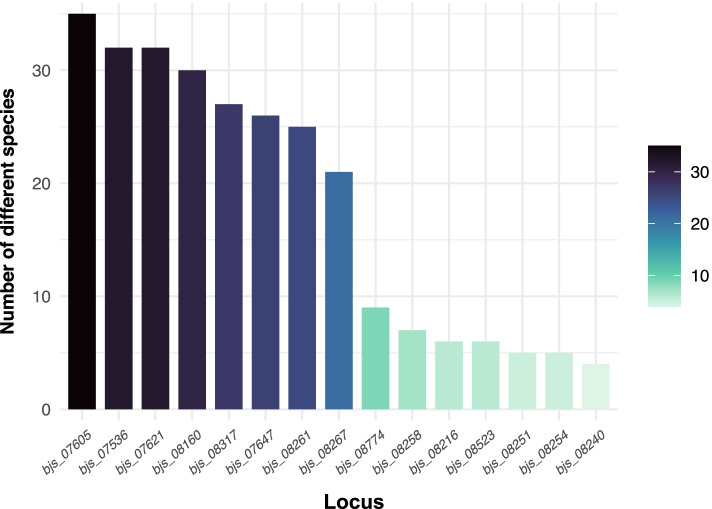
Distribution of the selected hypothetical proteins and of the *aspC* gene of *Bradyrhizobium japonicum* strain SEMIA 5079 (= CPAC 15) in other genomes of *Bradyrhizobium* species and strains not classified at species level

First, we analyzed the number of HPs retrieved against *Bradyrhizobium* genomes deposited in the NCBI database. We chose a threshold of ≥ 70% of identity and ≥ 50% of coverage for BLAST searches. The HPs set was split in two sets, the first composed of HPs broadly distributed within the *Bradyrhizobium* genus, and the second restricted to few species (Fig. 2; Supplementary File S1). Within the first group, the BJS_07605 protein proved to be the most broadly distributed member in *Bradyrhizobium*, detected in 35 different described species and 50 *Bradyrhizobium* spp. strains, totaling 159 proteins, followed by BJS_7536, which is present in 32 described species and 53 *Bradyrhizobium* spp. strains, totaling 155 proteins. In contrast, BJS_08240 is present in only four described species in addition to five *Bradyrhizobium* spp., totaling 16 proteins. Other examples of hypothetical proteins present in a few species are the BJS_08254, with five species and six *Bradyrhizobium* spp., with total of 17 proteins; and BJS_08251, present in five species and five *Bradyrhizobium* spp., summing 17 proteins.

The analysis of the distribution of hypothetical proteins in different species was also carried out. We observed that at least one strain of *B. japonicum* and of *B. diazoefficiens*, were present in all 15 proteins selected (14 HPs and *aspC*), followed by strains of *B. ottawaense* (14 proteins) *B. elkanii* (12 proteins). In contrast, *B. betae*, *B. guangxiense*, *B. guangzhouense*, *B. mercantei*, *B. neotropicale*, *B; rifense*, and *B. symbiodeficiens* showed only one protein. On average, species showed from five to seven hypothetical proteins of the set of 15 genes selected (Supplementary File S1).

Following with the analysis, eight strains of *Bradyrhizobium* spp. showed each one 14 out of the 15 proteins: three strains of *B. diazoefficiens* (H12S4, HH15, and XF7); *B. ottawaense* USDA 4; and four strains of *Bradyrhizobium* spp., CCBAU 15,615, CCBAU 15,635, CCBAU 43,298, SG09. Some variance regarding the proteins retrieved from *B. japonicum* strains was observed. While USDA 38 and J5 strains showed 13 proteins, CCBAU 25,435 carried 12 HPs, other *B. japonicum* strains (E109, FN1, NBRC 14,783, USDA 6, and SEMIA 5038) showed nine proteins. In contrast, 44 strains showed only one out of the 15 proteins. For example, *B. mercantei* strain SEMIA 6399 carried only the BJS_8160 protein. Similarly, strains CCBAU 51,670, CCBAU 53,424, CCBAU 53,426 of *B. guangzhouense* carried only BJS_08267. As both species do not nodulate soybean, we may suggest that the absence of HP might be explained because the symbiosis islands have been adapted to specific host plants. Other examples of strains showing only one protein were: *B. neotropicale* strain BR 10,247 (BJS_07536), *B. symbiodeficiens* strain 65S1MB (BJS_08160), and *B. rifense* strain CTAW71 (BJS_08160).

### Functional annotation of hypothetical protein-coding genes from symbiosis island

Several bioinformatics tools and resources were used to assign functions to the 14 HPs and to confirm the annotation of the *aspC* gene of the symbiosis island of SEMIA.

5079. The tools included Pfam, UniProtKB, InterPro, SMART, and CDD databases and all the results are shown in Supplementary File S2. Functional annotation was confirmed when conserved domains and similar functions were assigned in three or more databases, and allowed inference to 12 out of the 14 HPs (Table 1). For two HPs (BJS_08317 and BJS_08774), although protein domains were identified in the Pfam, the results were not significant. In this case, we annotated these domains as a first attempt to assign functions and understand their roles.

According to the functional annotation, 11 HPs were assigned as enzymes playing roles in different pathways, such as in the metabolism of hopanoid, methionine, queuosine biosynthesis, oxidation-reduction, and proteolysis. The majority of the enzymes were categorized as peptidases and hydrogenases. Two HPs were identified as membrane transporters, one as a DNA binding protein and another as a transcription regulator (Table 1). In addition, as many of the HPs were identified as enzymes, the physicochemical parameters as pI (isoelectric point), extinction coefficient, grand average of hydropathicity (GRAVY), and 3D structures were predicted and are summarized in Supplementary File S3. Most of the pI (isoelectric point) values calculated were around 6.0 to 6.5, with higher values for BJS_08267 (9.44), BJS_08774 (9.08) and BJS_08160 (8.98). The pI values are helpful to estimate where the protein will be localized on a 2-D gel. We also predicted the cellular localization of the HPs, the majority in the cytoplasm (Table 1). However, the trypsin-like peptidase (BJS_08254) was predicted as a non-classical secreted protein by Secretome P. As expected, the transporters were located in the membrane, the porin protein (BJS_07647) in the outer membrane, and the MFS transporter on the inner membrane (BJS_08267).

Using the EffectiveDB database, we analyzed the HPs that could be related to virulence and competitiveness, by investigating type III secretion signals, conserved binding sites of type III chaperones, type IV secretion peptides, eukaryotic-like domains, and subcellular targeting signals in the host (Table 2). The peptidase U49 (BJS_08240) was predicted as a potential virulent protein by the VICMpred tool. The trypsin-like peptidase (BJS_08254) and the acetylcholinesterase (BJS_08774) were found to be virulent by the VirulentPred tool. The remaining HPs were predicted as related to information and storage and also to metabolic or cellular processes. Interestingly, the trypsin-like peptidase (BJS_08254) was predicted as a potential T3SS effector using EffectiveDB database, corroborating with the VirulentPred tool. Furthermore, a glutathione-dependent formaldehyde-activating (GFA) enzyme (BJS_07621), as well as a pyridoxal phosphate-dependent aminotransferase (*aspC* gene, corresponding to BJS_08258) were predicted as potential T3SS effectors.

**Table 2:** Prediction of virulence factors and protein secretion systems in the 14 hypothetical and the AspC proteins of the symbiosis island of *Bradyrhizobium japonicum* strain SEMIA 5079 (= CPAC 15) annotated in this study

Protein ID	VICMpred	VirulentPred	EffectiveDB			
			**EffectiveT3**	**EffectiveCCBD**	**T4SEpre**	**EffectiveELD**
BJS_07536	Information and storage	Non-Virulent	-	-	-	-
BJS_07605	Cellular process	Non-Virulent	-	-	-	PF08267/PF01717
BJS_07621	Cellular process	Non-Virulent	1 (high confidence)	-	-	PF04828
BJS_07647	Metabolism Molecule	Non-Virulent	-	-	-	-
BJS_08160	Metabolism Molecule	Non-Virulent	-	-	-	PF02735
BJS_08216	Metabolism Molecule	Non-Virulent	-	-	-	-
BJS_08240	Virulence factors	Non-Virulent	-	-	-	-
BJS_08251	Cellular process	Non-Virulent	-	-	-	PF13365
BJS_08254	Cellular process	Virulent	1 (high confidence)	-	-	PF13365
BJS_08258 (AspC)	Metabolism Molecule	Non-Virulent	1 (high confidence)	-	-	PF00155
BJS_08261	Metabolism Molecule	Non-Virulent	-	-	-	PF00171
BJS_08267	Metabolism Molecule	Non-Virulent	-	-	-	PF07690
BJS_08317	Information and storage	Non-Virulent	-	-	-	-
BJS_08523	Cellular process	Non-Virulent	-	-	-	-
BJS_08774	Cellular process	Virulent	-	-	-	-

### Phylogeny and genes enrichment in host-bacteria interactions based on ELD analysis

We predicted secreted proteins based on eukaryotic-like domains (ELD) through the EffectiveELD tool (Table 2). These domains occur in eukaryotic genomes and show higher frequency in host-associated bacteria (pathogen or symbiont) genomes than in non-host-associated bacteria, indicating that they might play roles in the bacteria-host plant interaction. Some proteins were present in few bacterial genomes (Supplementary File S4). For example, the protein assigned as Met5 enzyme (BJS_07605) was present in six proteins showing the same domain in both *Bradyrhizobium icense* LMTR 13 and *Pseudonocardia acacia*, isolated from roots of *Acacia auriculiformis*. Two trypsin-like peptidases (BJS_08251 and BJS_08254) were present in eight bacterial species, including *B. elkanii* USDA 76 (16 proteins showing this domain). Surprisingly, the GFA enzyme (BJS_07621) and the non-homologous end-joining protein Ku (BJS_08160) showed several proteins with their domains in species of the Order Rhizobiales. The remaining proteins identified with the EffectiveELD tool, pyridoxal phosphate-dependent enzyme (BJS_0858), succinate-semialdehyde dehydrogenase (BJS_08261), and MFS transporter (BJS_08267) were present in the nitrogen-fixing *Paraburkholderia nodosa* strain DSM 21,604, but also in pathogenic *Burkholderia*. *Bradyrhizobium* species were not enriched of the last three proteins. GFA enzyme showed several proteins with GFA domain in different strains of *Bradyrhizobium* (Supplementary File S4). For example, 15 proteins in the SEMIA 5079 genome, 16 proteins in *B. diazoefficiens* SEMIA 5080, and 14 proteins in both *B. elkanii* USDA 76 and *B. icense* LMTR 13. Several proteins were also present in several other rhizobial species and strains of the genera *Bradyrhizobium*, *Rhizobium* and *Mesorhizobium*.

Following the criteria of threshold of ≥ 70% of identity and ≥ 50% of coverage for BLAST searches, we performed the phylogenetic analysis of all the 14 HPs and of the BJS_08258 (AspC) (Supplementary File S5). Our objective was to verify if there was a relationship with strains symbionts of soybean, and in general they were clustered together in all analyses. Interestingly, there were HPs as BJS_08254 not present either in *B. japonicum* type strain USDA 6 or in *B. diazoefficiens* type strain USDA 110, considering BLAST of both nucleotides and proteins, but other soybean symbionts were detected.

**Fig. 3:**
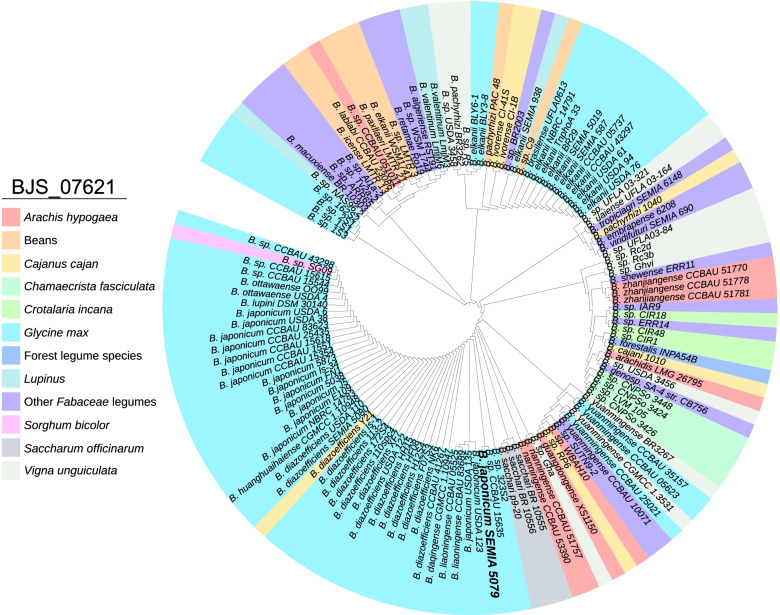
Phylogenetic inference of GFA proteins of *Bradyrhizobium* strains showing similarity with BJS_07621 of *Bradyrhizobium japonicum* SEMIA 5079 (= CPAC 15) and the relation with their host plants. *B.* sp. refers to strains not classified at the species level, only as *Bradyrhizobium* sp

We chose to explore in more details the evolutionary relationships and functions of the GFA, since this protein showed higher number of copies compared to the Ku protein (Supplementary File S4). Therefore, we searched for GFA proteins in *Bradyrhizobium* spp. genomes deposited in NCBI, selecting proteins with identity ≥ 70% (Supplementary File S1). A phylogenetic tree relating bacteria with their host plants was built (Fig. 3). *B. japonicum*, including strain SEMIA 5079, and *B. diazoefficiens* were clustered together. In the same clade, strains of *B. ottawaense*, *B. liaoningense* and *B. lupini* were also present. In addition, the cluster included *Bradyrhizobium* sp. SG09, isolated from sorghum (*Sorghum bicolor*) and *B. diazoefficiens* Y21, isolated from a root nodule of common bean (*Phaseolus vulgaris*) in China, but there was no information about their ability to nodulate and fix nitrogen with soybean. All the remaining strains in this cluster are reported as capable of nodulating soybean. Interestingly, other species reported as nodulating soybean plants were closer to symbionts of other legumes instead of *B. japonicum* or *B. diazoefficiens*. For example, *B. yuanmingense* strains were grouped with strains associated with cowpea (*Vigna unguiculate*) and peanut (*Arachis hypogaea*). Also, almost all *B. elkanii* strains (except for BLY3-8, BLY6-1, and SEMIA 938) were grouped in the same clade and were closer to symbiotic strains of common bean (*Phaseolus vulgaris*), and pigeon pea (*Cajanus cajan*). Strains associated with other beans, such as lima bean (*Phaseolus lunatus*), hyacinth bean (*Lablab purpureus*) and least snout bean (*Rhynchosia minima*) were grouped together, and were closer to other legumes, as *Retama monosperma* and *Stylosanthes viscosa*. In general, the results indicated that GFA proteins are present in several species associated with a large range of legume plants, with low host specificity.

**Fig. 4:**
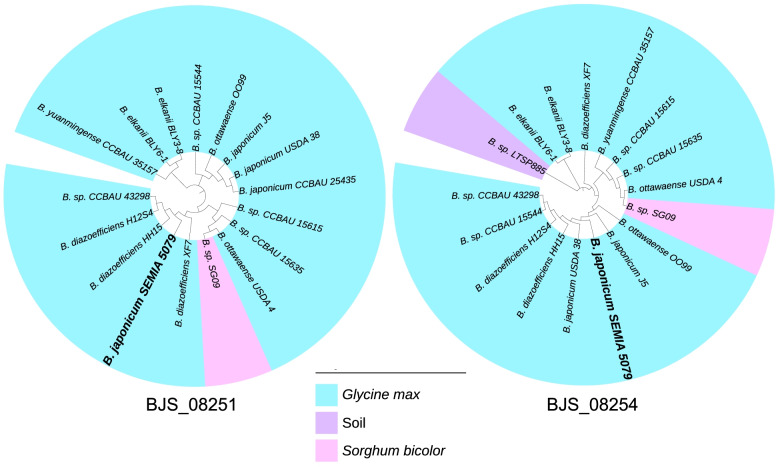
Phylogenetic inference of trypsin-like peptides of *Bradyrhizobium* strains showing similarity with BJS_08254 and BJS_08251 of *Bradyrhizobium japonicum* SEMIA 5079 (= CPAC 15) and the relation with their host plants. *B.* sp. refers to strains not classified at the species level, only as *Bradyrhizobium* sp

We also evaluated the phylogenetic trees of the two trypsin-like peptidases, since both were enrichment in bacteria-plant interactions by ELD analysis (Fig. 4). In both trees, the majority of the trypsin-like peptidases were identified in species nodulating soybean. In BJS_08251, only two proteins were retrieved from strains isolated from sorghum, while in BJS_08254 one protein was identified in a strain from sorghum and another was isolated in a soil under free-living conditions.

The expansion of these genes symbiotic genomes compared to non-symbiotic represents additional evidence of potential roles in the symbiotic process. While some proteins are broadly distributed in *Bradyrhizobium* species nodulating several legumes, as GFA and Ku proteins, indicating low host specificity, others show host specificity, such as the trypsin-like peptides, and may play roles in the *Bradyrhizobium*-soybean interaction.

### Gene expression levels of HPs in presence of genistein

**Fig. 5:**
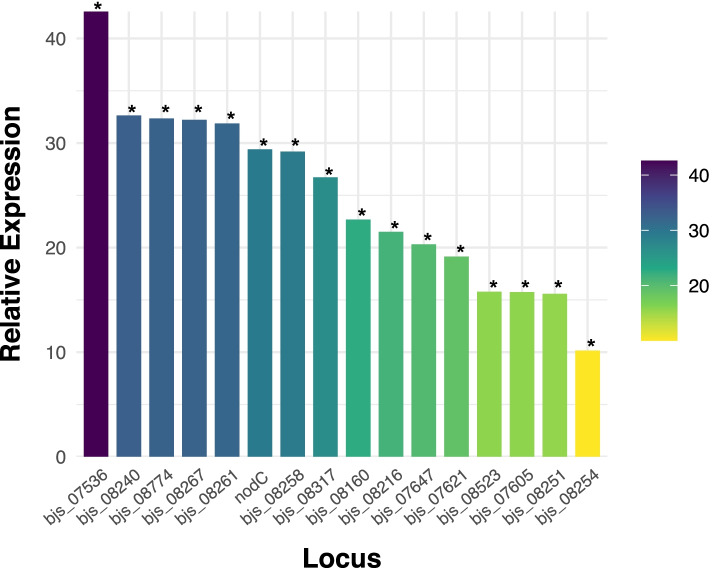
Expression levels of the hypothetical protein-coding and of *aspC* (*bjs_08258*) genes localized in the symbiosis island of *Bradyrhizobium japonicum* strain SEMIA 5079 (= CPAC 15) when induced by the *nod*-gene inducer genistein. Expression data shown for each gene represent the average of three biological replicates, with each one with three technical replicates. Data were normalized in relation to the endogen control (16 S rRNA gene). ^*^All genes obtained an expression statistically significant at the 5% level determined by REST2009 software

In the next step, we validated the relative expression levels of all 15 selected genes by quantitative RT-PCR. Significant expression levels were detected in all 15 genes after the induction with 2.5 µM of genistein. The induction levels ranged from 10.15- to 42.58-fold (Fig. 5). The expression levels of the *nodC* gene, used as a positive control were of 29.4-fold. The levels of the squalene-hopene cyclase (*bjs_07536*) were higher than the other 14 genes. Similar expression level patterns, ranging from 26.72 to 32.64-fold were observed in the set composed by HxlR-type HTH transcription regulator (*bjs_08317*), pyridoxal phosphate-dependent enzyme (*bjs_08258,=aspC*), succinate-semialdehyde dehydrogenase (*bjs_08261*), MFS transporter (*bjs_08267*), acetylcholinesterase (*bjs_08774*), and peptidase U49 (*bjs_08240*).

In contrast, other genes showed lower gene expression levels than the group described above. This group included both trypsin-like peptidases, *bjs_08254* and *bjs_08251*, with 10.15-fold and 15.58-fold, respectively, and the *met5* gene (*bjs_07605*) and 7-cyano-7-deazaguanine synthase (*bjs_08523*), with about 15.80-fold.

To get more insights about gene expression, we identified RpoN and RpoD-like promoters on the SEMIA 5079 genome through a list of promoters predicted in *B. japonicum* USDA 110. From the initial set of 1,210 RpoN-like promoters, 910 were identified, with 199 RpoN-like promoters identified in the symbiosis island; from the initial set of 4,007 RpoD-like promoters, 2,683 were identified in SEMIA 5079, with 481 located in the symbiosis island. It is worth to mentioned that several USDA 110 promoters aligned into the same regions in SEMIA 5079 and for those, only one promoter was counted.

RpoN-like promoters upstream of the *bjs_08317* and *bjs_07605* genes were identified (Fig. 1). Interestingly, for *bjs_07536*, a RpoN-like promoter was located upstream of a cluster of genes (*P450* and *fpps* genes), in which, bjs_07536 apparently belongs. Several RpoN-like promoters were identified in the region of *nod* and *fix* genes (close to *bjs_07647* and *bjs_07621*). For instance, a RpoN-like promoter upstream of *nodD1*, which is followed by the *nod* box genes. Also, other RpoN-like promoters were found upstream of *fixR* and *fixA* genes. For the *bjs_08774* region, a RpoN-like promoter was predicted upstream the *nodM* gene; however, a potential operon of three HPs, which contains the *bjs_08774* could be observed preceded by a RpoN-like promoter. RpoD-like promoters were predicted upstream of the *bjs_07647*, and *bjs_07621* (which contain multiple RpoD and RpoN promoters in the region). For the following genes, *bjs_08261*, *bjs_08267*, *bjs_08251*, *bjs_08216*, *bjs_08254*, and *aspC* (*bjs_08258*), neither RpoD or RpoN-like promoters were identified in their regions. While for *bjs_08240*, *bjs_08160*, and *bjs_08523*, RpoD-like promoters were predicted between genes upstream of their location and no clear operon organization was observed between those genes. In general, as expected, more RpoD-like promoters were identified than RpoN, in special in the *bjs_07647*, *bjs_07621* and *bjs_07605* gene regions. All the RpoN and RpoD-like promoter sequences predicted in SEMIA 5079 and their location are provided in Supplementary File S6.

## Discussion

### Genes coding for several enzymes are induced by genistein and might play roles in nitrogen fixation, competitiveness and environmental stress tolerance in SEMIA 5079

We submitted our sequences to several protein databases and bioinformatic tools that have been used to assign functions to hypothetical proteins in bacterial species, including *Bradyrhizobium* spp. [34, 35], creating a helpful workflow for functional annotation and curation. For 13 HP proteins, we found robust information in multiple databases, especially regarding known protein domains, which are described as highly conserved structure during the evolution process [36]. Although we were unable to find significant protein domains in the two remaining HP proteins, we continued to investigate their possible functions.

We found 11 HP proteins as potential enzymes, and they were classified in six classes. Transferases and hydrolases, each one containing four and three HPs, respectively, another protein was categorized as lyase, one as oxidoreductase, one as hydrogenase (chaperone), and one as ligase. Enzymes play several critical roles in rhizobia, being necessary for bacterial survival and nutrition [37], nodulation process [38], adaptation to stressful environments [39], competitiveness [40], growth in free-living conditions [30], physiological and metabolic processes [41], and virulence ability [33]. Saprophytic ability and competitiveness are important features to a successful symbiosis and in SEMIA 5079 these genes are located in the symbiosis island.

### Transferases

Transferases catalyze the transfer of a specific group from one substance to another [42], and we identified four transferases (BJS_07605, BJS_08258, BJS_08523, and BJS_08774). BJS_07605 was predicted to be a 5-methyltetrahydropteroyltriglutamate-homocysteine methyltransferase (MetE protein; EC 2.1.1.14), belonging to the UROD/MetE-like superfamily. MetE protein has been reported as involving in methionine (cobalamin) biosynthetic pathway in soil bacteria as *Pseudomonas putida* [43], and *Saccharopolyspora spinosa* [44]. In rhizobia species, *metE* has been identified in transcriptomes studies in both bacteroids and free-living conditions in USDA 110 [35, 36]. In the plant pathogen *Ralstonia solanacearum*, *metE* is under the control of the pathogenicity regulator HrpG, and *metE* mutants showed reduced disease symptoms on tomato (*Solanum lycopersicum*) [45]. The *metE* has also been related to stress conditions tolerance, e.g. to acetate and temperature in *Escherichia coli* [46], and copper in yeast *Rhodotorula mucilaginosa* [47]. Thus, we may suggest that the main roles of this gene in SEMIA 5079 may be the tolerance to environmental stress conferring higher saprophytic ability, in addition to competitiveness. Roles in BNF have also been proposed. The disruption of the *metH* (cobalamin-dependent enzyme gene) in *S. meliloti* causes inability of fix nitrogen in alfalfa plants. The expression of *metE* in *metH* mutants complements the Fix^−^ phenotype, as the average plant height, nitrogenase activity and the percentage of pink nodules are dramatically improved [48].

The BJS_08258 protein was predicted as an aminotransferase class I/II-fold pyridoxal phosphate (PLP)-dependent enzyme, also named aspartate aminotransferase (AspC protein, EC 2.6.1.1). Almost all PLP-dependent enzymes are involved in biochemical pathways with amino compounds, mainly amino acids [49]. In *S. meliloti* 4R3, alteration in aspartate transaminase resulted in mutants unable to catabolize aspartate and to induce effective nodules on *M. sativa* [50, 51]. The *aspC* gene was found expressed in nitrogen-fixing bacteroids in the *S. meliloti*-*Medicago truncatula* symbiosis [52]. Also, an *aspC* mutant of *S. meliloti* showed a nitrogen-starvation phenotype during plant infection assays and significantly reduction in nitrogenase activity [53]. Finally, an aspartate aminotransferase was identified as differentially expressed in the presence of genistein in *B. japonicum* SEMIA 5079 [54], supporting our data that AspC is induced by genistein in this strain and might play a role in BNF.

The BJS_07536 was predicted as a squalene-hopene cyclase (Shc protein, EC 5.4.99.17) involved in the hopanoid biosynthesis pathway (secondary metabolite biosynthesis) [55]. Hopanoids are lipids that are mainly located in bacterial membranes, associated with membrane fluidity, lipid rafts, and stress tolerance [56]. Many hopanoid-producing bacteria are capable of fixing nitrogen, including *Bradyrhizobium* spp. [57, 58], and it has been suggested that they facilitate nitrogen metabolism, what could explain the highest gene expression level of this gene. In photosynthetic *Bradyrhizobium* sp. strain BTAi1, an *shc* mutant showed slower-growing under optimal growth conditions, lower resistance to stresses, and reduced ability to survive intracellularly in its host plant *Aeschynomene* [59].

In general, transferases play roles in spore germination, synthesis of lipoproteins, and virulence in bacteria [60, 61]. Corroborating with that, in our study, two transferases, BJS_07605 (MetE) and BJS_07536 (Shc) were also associated with stress tolerance and virulence in bacteria species, while BJS_08258 (AspC) was associated with an effective nitrogen fixation process.

### Hydrolases

Hydrolase enzymes catalyze the addition of water to a substrate through a nucleophilic substitution reaction and are the biocatalysts most commonly used in organic synthesis [62]. Hydrolases play essential roles in the invasion of the host tissue, escaping host defense mechanisms [63]. In our study four proteins were predicted as hydrolases. Three of them are peptidases, BJS_08251 and BJS_08254 predicted as putative trypsin-like peptidases belong to serine peptidases family S1, and BJS_08240 predicted as peptidase U49. BJS_08774 carries an acetylcholinesterase domain. In our results, BJS_08254 was predicted as secreted, a virulence factor, a potential T3SS effector, and enrichment by ELD tool. We may thus suggest that this trypsin-like peptidase could act in the infection process of soybean by *Bradyrhizobium*, as the protein was majorly identified in *Bradyrhizobium* symbionts of this legume (Fig. 4).

Trypsin peptidases (STS) have been described as markers in phytopathogenic fungi genomes [64]. Secretomes of plant pathogens are enriched with endopeptidases [65], and mutations lead to the loss of virulence ability [66, 67]. Therefore, in *Bradyrhizobium* these proteins could facilitate the host plant-bacteria interaction. STS was identified among the secreted proteins in *B. diazoefficiens* USDA 110 under free-living conditions [68] and in bacteroids [69], but there is little information about their roles in the infection process. Serine protease DO-like proteins were identified on the secretome of *R. etli* CE3 [70], and in the proteomic analysis of *Rhizobium favelukesii* LPU83 in response to acid stress [71]. Also, extracellular serine proteases were identified in the genomes of *Rhizobium* strains tolerant to high temperatures [72], and mutations in the serine protease (CtpA protein) lead to increased sensitivity to desiccation in *R. leguminosarum* [73]. In the *S. meliloti-M. truncatula* symbiosis, a peptidase (HrrP) cleaves host-encoded signaling peptides and mediates symbiotic compatibility [74]. Little information is available about the functions of peptidases U49. Besides that, roles on microbial virulence and pathogenesis have been proposed [75]. We may then hypothesize the potential roles of these STS in SEMIA 5079 both in the infection process and stress tolerance.

### Lyase, ligase, oxidoreductase and hydrogenase

The BJS_07621 protein was predicted as a lyase, a glutathione-dependent formaldehyde-activating (GFA) enzyme (Gfa protein, EC 4.4.1.22), of the glutathione-dependent formaldehyde oxidation pathway, involved in C1 metabolism and methanol oxidation in *B. diazoefficiens* [76]. In *B. diazoefficiens* USDA 110, *gfa* was induced in cells grown with vanillin (aromatic compound) as sole source of carbon [77], in *M. loti* MAFF303099 in response to salt stress [78], in *R. tropici* CIAT 899 to acid stress [79], and in USDA 110 cells exposed to elevated atmospheric CO_2_ [80]. Therefore, the enzyme has been associated with bacterial growth under different environmental stresses, and the several copies found in SEMIA 5079 might indicate advantages under stressful conditions.

The BJS_08261 protein was identified as an oxidoreductase enzyme, a succinate-semialdehyde dehydrogenase (GabD protein, EC 1.2.1.24) involved in the GABA (γ-aminobutyric acid) metabolism [81]. When *R. leguminosarum* bv. viciae strain 3841 was mutated in aminotransferase and succinate semialdehyde dehydrogenases, the mutants were still able to fix nitrogen on pea plants [82]. In *R. etli*, *gabD* is required for glutamine utilization, and mutations affected growth in culture medium with glutamine as only carbon source [83]. Proteomic analysis showed that GabD protein was increased in *R. leguminosarum* SRDI565 cultivated with sulfosugar sulfoquinovose (SQ) as a carbon source [84]. GabD proteins were identified in rhizobia proteomes, including *B. diazoefficiens* USDA 110 cells grown in HM (Mueller-Hinton) medium [85], *R. tropici* CIAT 899 induced by apigenin and salt stress [86], and *R. favelukesii* LPU83 in response to acid stress [71]. In an association mapping study (GWAS) using 153 strains of *S. meliloti* to map regions associated with symbiosis phenotypes [87], two of the reported genes (*gabD* and *queC*) were annotated and validated in our study. Therefore, GabD in SEMIA 5079 may be associated with the utilization of different carbon sources and also with tolerance to environmental stresses.

The BJS_08216 protein was predicted as the hydrogenase maturation factor HypA, a nickel metalloenzyme, an accessory protein for nickel incorporation into hydrogenases [88]. The *hypA* gene is part of hydrogenase uptake *(hup)* operon located in the symbiosis island of several rhizobia, such as SEMIA 5079 [6], SEMIA 5080 [6], USDA 110 [89], *R. tropici* CIAT 899, and PRF 81 [90]. Hydrogen-uptake genes improve symbiotic efficiency in several legumes, including soybean [91]. However, often the operon is not complete and functional. *B. diazoefficiens* USDA 110 has two sets of the *hup* gene cluster, a complete set of functional *hup* genes located outside the symbiosis island and an incomplete set of non-functional *hup* genes within the symbiosis island [6]. In *B. japonicum* USDA 6 and SEMIA 5079 the genes are duplicated and the operon incomplete, or are pseudogenes; therefore, also non-functional [6, 89]. In addition, a Hup^−^ phenotype has been reported in *B. japonicum* SEMIA 5079 [92]. In *R. leguminosarum* a mutation in *hypA* confirmed that the gene is essential for hydrogenase activity, as it is required for the correct processing of the large hydrogenase subunit [93]. Nevertheless, several *hyp* and *hup* genes, including *hypA*, were induced in USDA 110 cells during chemoautotrophic growth [94]. Similarly, *hypA* was expressed in SEMIA 5079 even though apparently the hydrogenase is not functional [94], what might indicate other possible functions.

The BJS_08523 protein was predicted as a 7-cyano-7-deazaguanine synthase, QueC protein (EC 2.7.7) involved in the biosynthesis pathway of queuosine [95]. Mutations in *queC* of *S. meliloti* resulted in ineffective BNF [96], and in a GWAS study performed to identify BNF traits in *S. meliloti, queC* was identified in one of the regions mapped [87]. In *Shigella flexneri*, full expression of the virulence VirF was shown to require queuosine Q34 [97]. Therefore, in SEMIA 5079 the *queC* gene might be relative both with the infection process and the effectiveness of the BNF process.

### Transporters and other proteins

The BJS_07647 was predicted as a porin, a class of proteins that are β-barrel channels located in Gram-negative bacterial outer membranes and allow the diffusion of different molecules, being also associated with multidrug-resistant [98]. Environmental conditions such as water availability, oxidative stress, heavy metals, temperature, and nutrients shortage affect the expression and/or activity of porins. [99]. In rhizobia, there are studies relating porin to increased expression in bacteroids of *Bradyrhizobium* sp. (*Lupinus*) in plants treated with high doses of glyphosate doses [100], required for USDA 110 cells growth under manganese deficiency [101], and involved in copper transport in *R. etli* CFN4 [102, 103]. Also, porins were identified in proteomic and transcriptomic studies of cells and bacteroids of USDA 110 [68, 69, 92]. In our results, BJS_07647 was predicted as an outer membrane, as expected to porins, and we may attribute roles in tolerance to different toxic compounds, as the high manganese and aluminum levels of many Brazilian Cerrado soils.

The BJS_08267 was predicted as a major facilitator transporter (MFS), a superfamily composed of multidrug resistance efflux pumps (MDR). Besides the roles in multidrug resistance, the genes of this family have been primarily described in bacteria and might play a general role in detoxification of toxic compounds and in rhizobia actuating in competitiveness in the rhizosphere [104]. MFS transporters have been described in USDA 110 involved in nitrate assimilation and nitric oxide detoxification [105], and in *Rhizobium* sp. RC1, mediating the uptake of haloacids into the cells [106]. In relation to the symbiosis, MFSs have been described contributing to nodulation competitiveness in *S. meliloti* [107] and in *R. leguminosarum* bv. viciae [108]. Thus, this could also be another protein facilitating detoxification of toxic compounds and competitiveness in SEMIA 5079.

The BJS_8160 was identified as a non-homologous end-joining protein Ku (Ku protein); these proteins are specialized in repairing DNA damages [109] In *S. meliloti* Ku proteins have been identified in both free-living conditions and bacteroids [110], and related to stress conditions, as heat and nutrient starvation [111]; however, in *S. meliloti*, Ku protein was not essential to the symbiotic interaction with *M. truncatula* [112]. Our results indicate enrichment of this Ku protein in rhizobial species and we may suggest a role in SEMIA 5079 in repairing DNA damages derived from stressful conditions, such as high temperatures and acidity.

A probable HxlR-type HTH domain, present in putative transcription regulators with a winged helix-turn-helix (wHTH) structure, was found in BJS_08317. This DNA-binding protein acts as a positive regulator of the formaldehyde-inducible *hxlAB* operon in *Bacillus subtilis*, which is part of the ribulose monophosphate pathway involved in formaldehyde fixation [113]. HxlR transcriptional regulators have been induced in other bacteria, such as *P. fluorescens* in the presence of heavy metals [114], *Lactobacillus acetotolerans* in response to ethanol stress [115], and *Bacillus atrophaeus* UCMB-5137 stimulated by maize root exudates [116]. As other enzymes identified in our study, BJS_08317 could be associated with the saprophytic ability of SEMIA 5079, since this protein has been associated with bacterial survival in response to different compounds.

### Expression levels of hypothetical protein-coding genes located in symbiosis island in presence of genistein

We applied some parameters to choose the hypothetical protein-coding genes for further functional annotation, followed by confirmation by qRT-PCR. Firstly, to identify potential new genes related to BNF traits, we selected genes from the symbiosis island of SEMIA 5079, which contains several predicted hypothetical protein-coding genes [6]. As the main genes related to BNF, such as *nod*, *fix*, and *nif* genes and are positioned in symbiosis islands in *Bradyrhizobium* and some *Mesorhizobium* strains [117–119], we selected the hypothetical protein-coding genes close to these genes, with an emphasis on those related to nodulation. In rhizobia, *nod* genes are usually organized in operons, as *nodABC* in *S. meliloti* [120] and *nodYABCSUIJnolMNO* in USDA 110 [121], indicating that their expression regulation involves common mechanisms, including induction by flavonoids and common transcription factors [122, 123]. Therefore, the hypothetical protein-coding genes selected in our study have high probability of being under the same regulation as the nodulation or nitrogen fixation genes.

We quantified the gene expression levels of the 15 selected protein-coding genes in the presence of 2.5 µM of genistein, a known inducer of nodulation genes in *Bradyrhizobium* [7, 8], and confirmed expression for all of them in cells induced at the initial exponential phase, an approach reported in previous studies [28, 32]. For example, the porin gene (*bjs_07647*) was close to several nodulation genes previously identified as induced by genistein, as *nodC* in both *B. japonicum* SEMIA 5079 (1 µM) [31] and SEMIA *B. diazoefficiens* 5080 (5 µM) [35], and also close to *nodD1*, *nodD2* and *nodA* genes, all induced in *B. liaoningense* CCBAU05525 (0.5 µM) [32]. Another example is the HxlR transcriptional gene (*bjs_08317*), close to the *nopP* gene, which was also previously induced by genistein in SEMIA 5079 [31]. Higher expression levels were detected in five HP genes (*bjs_07536-*hopanoid biosynthesis pathway; *bjs_08240-peptidase*, *bjs_08774-* acetylcholinesterase, *bjs_08267-* MFS transporter and *bjs_08261-* succinate-semialdehyde dehydrogenase), reinforcing the hypothesis of major roles in BNF. These results also support our strategy of choosing genes close to known BNF genes, followed by a functional workflow of annotation and curation, and the transcription validation in the presence of a *nod*-gene inducer.

We predicted RpoN and RpoD-like promoters in SEMIA 5079 genome to get insights about gene expression regulation. We chose to select those types of promoters since RpoN promoters (σ^54^) have been identified as associated with nitrogen-fixing ability [124–126] and RpoD promoters (σ^70^) associated with free-living conditions [125–128]. Corroborating with the USDA 110 prediction, more RpoD than RpoN promoters were identified on SEMIA 5079 genome. We did not find any clear correlation between gene expression levels and promoter identification. Indeed, in five HPs genes, we did not find any RpoN and RopD-like promoters; however, three of them (*bjs_08261*, *bjs_08267* and *aspC*) are located close in the genome and showed high gene expression levels compared to the remained genes. The *bjs_08267* gene (MFS transporter) was preceded by several transporters-coding genes (Fig. 1), while *bjs_08261* (GabD) and *aspC* are enzymes associated with biosynthesis and oxidation process, with roles in bacterial growth and BNF ability, respectively. Therefore, further investigation in order to identify other types of promoters regulating those genes should be performed. Also, other hypothesis could be the regulation of those genes by the two sigma-54 factors copies present in *B. japonicum* located in the symbiosis island: *bjs_08297* (rpoN1 protein, 46,402 to 47,898 bp) and *y4pA* gene (*bjs_08283*, 58,369 to 60,123 bp). These genes have been reporting regulating several genes with diverse functions, including genes related to nitrogen fixation, free-living conditions, and transport on rhizobia species genes [124, 129, 130].

Interestingly, the highest gene expression level was observed to *bjs_07536* (hopanoid biosynthesis pathway), which seems to belong to the P450 operon preceded by a RpoN promoter, as previously reported in *B. japonicum* USDA 110 [125, 130, 131] and identified in our study. Other genes as *bjs_08317* (HTH transcription regulator) and *bjs_07605* (*metE*) showed an RpoN promoter in their upstream region, and in special *metE* gene has its expression associated with the NifA-RpoN regulon in *M. loti* [132]. The genes, as *bjs_07647* (porin protein) and *bjs_07621* (GFA enzyme) were preceded by RpoD-like promoters, which makes sense, since both genes are associated with bacterial growth and tolerance to different environment stresses. In the *bjs_08160* gene region, which was annotated as a protein Ku associated with free-living conditions and bacteroids, only RpoD-like promoters were predicted. In a similar way, the *bjs_07647* (porin) region was predicted with a enrichment of RpoD-like promoters, thus, raising the idea the others HPs located in those regions could play important roles to free-living conditions in *B. japonicum* as *bjs_0764*7 and *bjs_08169.* In general, the prediction the RpoN and RpoD-like promoters in SEMIA 5079 was similar to the predictions in the symbiosis island of USDA 110 [126]. Also, in some of our HPs with high level of expression we are not able to find RpoN and RpoD-like promoters, raising perspectives of future studies to find other promoters regulating those genes and playing important roles in the BNF capacity, saprophytic ability, and tolerance to stresses. Finally, we also provided here an extensive list of predicted promoters for future functional studies in SEMIA 5079.

## Conclusions

The genome of *B. japonicum* strain SEMIA 5079 contains a large proportion of hypothetical protein-coding genes, especially in its main symbiosis island, a key region in the circular genome where the nodulation and nitrogen fixation genes are located. Our study brings a workflow of bioinformatics tools/databases to correctly assign the function of hypothetical protein-coding genes present in SEMIA 5079 symbiosis island and that may be applied to other rhizobia. Fourteen hypothetical protein-coding genes and another one later identified as *aspC* were studied in more detailed and were induced by the *nod*-gene inducer genistein, from 10.15- up to 42.58-fold. Some proteins were specific to *Bradyrhizobium*, while others were present in several rhizobia, and the analysis of orthologues helped to predict roles in BNF, including competitiveness and infectiveness. Although the results are descriptive and efforts should be done to identify the role of HP genes on symbiosis island, the study contributed to improve the knowledge about the genomics features of this outstanding strain used in more than 70 million doses of i soybeaninoculants yearlyin Brazil and in other countries of South America.

## Materials and methods

### Functional annotation of hypothetical protein-coding genes from symbiosis island

The annotated genome of *B. japonicum* SEMIA 5079 (CP007569.1 - GenBank) was retrieved from the NCBI Genome database [133]. Hypothetical protein-coding (HP) genes located in the symbiosis island of SEMIA 5079 were initially collected based on an automatic annotation of this genome [6]. All genes predicted inside the symbiosis island were retrieved into a unique FASTA format file. To choose HP genes located in the symbiosis island, some criteria were used: genes located close to others previously described as playing roles on the BNF process (e.g. *nod*, *fix*, *nif* genes), and with size range of 600 to 2,500 bp (avoiding truncated genes or pseudo-genes). The hypothetical protein-coding gene sequences were taken separately to assign their accurate function based on several bioinformatic tools.

To assign the best function to HP genes, we analyzed the chosen protein sequences against sequences deposited in several databases: PFAM 34.0 (http://pfam.xfam.org/); Kyoto Encyclopedia of Genes and Genomes v98.1 (KEGG) (http://www.genome.jp/kegg/); Universal Protein Resource Knowledgebase (UniProtKB) (http://www.uniprot.org/); Rhizobase (genome.microbedb.jp/rhizobase); NCBI-CDD v3.18 (https://www.ncbi.nlm.nih.gov/Structure/cdd/wrpsb.cgi); InterPro (https://www.ebi.ac.uk/interpro/); SMART (http://smart.embl-heidelberg.de/) and Gene Ontology – GO (http://geneontology.org/).

For each protein, physical and chemical parameters as molecular weight, theoretical pI, amino acid composition, isoelectric point, aliphatic index, grand average of hydropathicity, among others were performed with the ProtParam tool from Expasy server (https://web.expasy.org/protparam/). Sub-cellular localization of the HPs was predicted using online tools as TMHMM Server v. 2.0 (http://www.cbs.dtu.dk/services/TMHMM/); CELLO v.2.5 (http://cello.life.nctu.edu.tw/); PSORTb version 3.0.3 (https://www.psort.org/psortb/), and PSLpred (http://crdd.osdd.net/raghava/pslpred/). Presence of classical signal peptide in hypothetical proteins was predicted using SignalP-5.0 Server (http://www.cbs.dtu.dk/services/SignalP/), and for non-classical protein secretion the SecretomeP 2.0 Server (http://www.cbs.dtu.dk/services/SecretomeP/). The 3D modeling of hypothetical proteins was performed by the online serve SWISS-MODEL (https://swissmodel.expasy.org/).

In order to predict proteins related to soybean infection by *Bradyrhizobium*, prediction of virulence factors was performed using VICMpred (http://crdd.osdd.net/raghava/vicmpred/) and VirulentPred (http://203.92.44.117/virulent/index.html/) tools that are based on PSI-Blast and the support vector machine (SVM) method based on patterns, amino acid and dipeptide composition of bacterial protein sequences.

The occurrence of the studied hypothetical proteins in genomes of other strains of *Bradyrhizobium* was verified by similarity searches using BLASTX (E ≥ 1 × 10^− 6^, identity ≥ 70%), at the NCBI (https://blast.ncbi.nlm.nih.gov/Blast.cgi). Further, only sequences from Reference Sequence (RefSeq) database (http://www.ncbi.nlm.nih.gov/RefSeq/), which provides a non-redundant collection of sequences representing genomic data, transcripts and proteins were kept. Also, in cases where more than one protein per genome was identified, only the protein with more similarity with the hypothetical protein-coding gene from SEMIA 5079 was used. Protein alignments between HPs queries and the resulting protein sequences obtained from BLAST steps were performed using the CLUSTAL W program in MEGA version 11 [134]. Phylogenetic trees were constructed using the neighbor-joining (NJ) method [135, 136], with 1,000 bootstrap replicates.

We searched in SEMIA 5079 genome for both promoter motifs similar to σ^54^ RpoN-dependent promoters, mainly activated in symbiosis and promoter motifs similar to σ^70^ RpoD-dependent promoters, associated with free-living conditions. Through BLASTN, we searched on SEMIA 5079 genome for 1,201 RpoN-like promoters and 4,007 RpoN-like promoter sequences upstream of TSSs (transcriptional start sites) predicted in *B. japonicum* USDA 110. Only promoters located in intergenic regions were kept and analyzed.

### Bacterial strain, growth conditions and genistein essay

*Bradyrhizobium japonicum* strain SEMIA 5079 (= CPAC 15, =DF 24, =CNPSo 7), was obtained from the “Diazotrophic and Plant Growth Promoting Bacteria Culture Collection” of Embrapa Soja (WFCC Collection #1213, WDCC Collection #1054). The strain was cultured at 28 °C in modified-YM solid medium supplemented with Congo red (0.025%) [137]. Bacterial pre-cultures were grown under shaking (100 rpm) at 28 °C in 10 mL of TY broth [138]. When reaching OD_600_ of 0.4, 1 mL was inoculated into 100 mL of TY broth supplied with genistein at a final concentration of 2.5 µM, followed by incubation at 28 °C under shaking (100 rpm and 28 °C) for approximately 1 h, up to the exponential phase (OD_600_ 0.5 ~ 0.6). The cells were then recovered and used for RNA isolation and expression analysis. The experiment was performed with a randomized block design, with three biological replicates. The control treatment received only methanol, which was the solvent used to prepare the genistein solution.

### RNA isolation and cDNA synthesis

Total RNA was extracted using TRIzol^®^ reagent (Thermo Fischer Scientific Inc., Waltham, MA, USA) following the manufacturer’s instructions, with some modifications [30]. Thirty mL of cells were harvested by centrifugation (10,000 rpm at 4 °C for 10 min), the cell pellets were resuspended with 1 mL of NaCl solution (0.85%), the pellet solutions were transferred to new 2 mL microtubes and harvested by centrifugation as described above. Pellets were then resuspended in 300 µL of lysis buffer (250 µL TE 10:1; 40 µL SDS 10%; 10 µL lysozyme at 5 mg/mL) and incubated at 40 °C for 5 min. Following, 1.5 mL of Trizol was added to the samples, vortexed for 15 s, and incubated at room temperature (24 ± 1 °C) for 5 min. Cells were then centrifuged (10,000 rpm at 4 °C for 10 min) and the aqueous phase (~ 800 µL) was transferred to a new microtube with 250 µL of chloroform, vortexed for 15 s and incubated at room temperature (5 min). Cells were then centrifuged as described above, the aqueous phase (~ 400 µL) transferred to new microtubes, and the RNA was precipitated with 500 µL isopropanol. After 20 min on ice, RNA samples were collected by centrifugation at 14,000 rpm for 15 min and resuspended in ultra-pure water (50 µL). After that, 3 M sodium acetate and 250 µL of 100% ethanol were added to the RNA samples and mixed well by gently inverting the tubes several times. A new step of centrifugation was performed as described above, the upper phase was discarded and the pellets were washed with 500 µl ethanol 70%. The samples were centrifuged again, and RNA pellets were kept to dry at room temperature (24 ± 1 °C) for 30 min. Finally, the dry pellets were dissolved in 50 µL of water ultra-pure.

RNA concentration was estimated in a NanoDrop™ 8000 Spectrophotometer (Thermo Fischer Scientific Inc., Waltham, MA, USA) and integrity was assessed in 1.0% agarose gel by electrophoresis. RNA samples were treated with DNAse I (Thermo Fischer Scientific Inc., Waltham, MA, USA), according to the manufacturer’s instructions. Following, cDNA strands were synthesized from 5 µg of treated RNA using the SuperScript^→^ III First-Strand Synthesis System (Thermo Fischer Scientific Inc., Waltham, MA, USA) and random primers, according to the manufacturer’s instructions.

### Quantitative real-time PCR (qRT-PCR) approach

Primers were designed using the Primer Express 3.0^®^ software (Applied Biosystems, Foster). The default parameters for qRT-PCR were applied, with an amplicon range of 50–150 bp size. The primers sets were checked against SEMIA 5079 genome by BLASTN tool to verify their specificity, and the list of the primers used is shown in Table 3.

**Table 3:** Genomic position and primer sequences for the qRT-PCR assay of the 14 hypothetical and the *aspC* genes of the symbiosis island of *Bradyrhizobium japonicum* SEMIA 5079 (= CPAC 15) selected for this study

Locus	Protein ID	Physical Location	Primer Sequence ( 5’– 3’)		Amplicon bp size
*bjs_07536*	AHY48760	415,677.415,677 (+)	F GTGTCGACCAACATTCATGC R TGGGATACAGCCACGAAAC		142 bp
*bjs_07605*	AHY48686	318,489.320,720 (+)	F GGCAAGTTGGGTTCAGTTTG R CTTTGCGAACCGGTTATAGG		97 bp
*bjs_07621*	AHY48670	287,116.288,339 (+)	F GCGGCGCTATCAAATTCTAC R ACGAGCAAGTGGACAAATCC		100 bp
*bjs_07647*	AHY48641	259,705.260,430 (-)	F TTTCGTCATGCGTTGTGG R ATTTCCGTCCTCATGCGTAG		114 bp
*bjs_08160*	AHY56892	9,349,925.9,350,872 (+)	F CGATCCGCGATATCTCATTC R ATCGCGACCTTGTTCATCTC		111 bp
*bjs_08216*	AHY48554	152,594.154,168 (+)	F GATCCACGGGATGTTTGAAG R CGCAGATCAATTCACGTAGC		137 bp
*bjs_08240*	AHY48531	125,139.126,544 (-)	F CCTTCGAGGACTTGATTTCG R GACGTGTCCGTTGAATTCTG		107 bp
*bjs_08251*	AHY48520	98,439.100,538 (+)	F CAACGATGATACGGTTGCAG R GCATGACCTTGATTGCCTTC		131 bp
*bjs_08254*	AHY48517	96,080.96,979 (+)	F GGTCGGCTATTTCAAGATCG R GATAGCCCGAAATGTTGACG		74 bp
*bjs_08258* (*aspC*)	AHY48514	91,505.92,803 (+)	F ATCCGAGCTGCCATTACATC R GCAATGTTGTTCGGCTGTAG		91 bp
*bjs_08261*	AHY48511	85,326.86,360 (+)	F CGTGATCAATCTCGTCTTCG R TGAAGGAGACTTTCCGGATG		83 bp
*bjs_08267*	AHY48505	76,029.77,225 (+)	F TCCTGATCTGGATGTCAACG R AGAAGCATGGGATGAGCAAC		105 bp
*bjs_08317*	AHY48455	18,261.20,504 (+)	F AAATAGTCCGGCTGACAAGG R CATAGGTCTTCGTGCAATCG		102 bp
*bjs_08523*	AHY56944	9,425,196.9,426,470 (+)	F TGAGAAGCAGGAAGAGTTCG R CTCCGAAGGCGAGAAATATG		134 bp
*bjs_08774*	AHY56918	9,392,008.9,393,012 (+)	F ACAAAGGGTGGCTCGAAAC R TCGTAAGCGCGTAGTAAACG		138 bp

(**+**) strand positive, (-) strand negative, *F* primer forward, *R* primer reverse

Reactions were performed on a qRT-PCR thermocycler 7500 (Applied Biosystems), according to the manufacturer’s recommendations, using a Platinum SYBR Green qPCR SuperMix-UDG with ROX kit (Thermo Fischer Scientific Inc., Waltham, MA, USA). Initially, the PCR primers efficiency was determined using a quantitative standard curve for each pair of primers (10-fold serial dilution of a cDNA bulk containing all the biological replicates of both treatment and control). The Ct (cycle threshold) values obtained in each dilution were plotted as a function of logarithm of the cDNA dilutions, with the slope of regression curve used to estimate primer efficiency values [139]. All qPCR reactions were performed in 12.5 µL reactions containing 1 µL of cDNA, 6.25 µL of SYBR Green with ROX, 0.2 µM of each primer (reverse and forward), and 4.25 µL of ultra-pure water. The PCR cycling program consisted of 50 °C for 2 min, 95 °C for 10 min, followed by 45 cycles at 95 °C for 2 min, 60 °C for 30 s and 72 °C for 30 s. The reactions were performed with three technical replicates for each biological replicate. The calculation of the expression and the statistical analysis were carried out with the software REST 2009 [140]. The expression of the 16 S rRNA endogenous gene was used as an internal control for normalization [31, 32], and the genistein responsive *nodC* gene was used as positive control [31, 141].

## Supplementary Information

**Additional file 1.**

**Additional file 2.**

**Additional file 3.**

**Additional file 4.**

**Additional file 5.**

**Additional file 6.**

## Data Availability

All data are included in the manuscript and in the supplementary material section.

## References

[1] CONAB (2021). Acompanhamento da safra brasileira. Cia Nac Abast Acompan da Safra Bras.

[2] Hungria M, Mendes IC (2015). Nitrogen fixation with soybean: the perfect symbiosis?. Biological Nitrogen Fixation.

[3] Zilli JÉ, Alves BJR, Rouws LFM, Simões-Araujo JL, de Barros Soares LH, Cassán F (2020). The importance of denitrification performed by nitrogen-fixing bacteria used as inoculants in South America. Plant Soil.

[4] Hungria M, Franchini JC, Campo RJ, Crispino CC, Moraes JZ, Sibaldelli RNR (2006). Nitrogen nutrition of soybean in Brazil: Contributions of biological N_2_ fixation and N fertilizer to grain yield. Can J Plant Sci.

[5] de Carvalho FG, Selbach PA, Bizarro MJ (2005). Eficiência e competitividade de variantes espontâneos isolados de estirpes de *Bradyrhizobium* spp recomendadas para a cultura da soja (*Glycine max*). Rev Bras Cienc Solo.

[6] Siqueira AF, Ormeño-Orrillo E, Souza RC, Rodrigues EP, Almeida LGP, Barcellos FG (2014). Comparative genomics of *Bradyrhizobium japonicum* CPAC 15 and *Bradyrhizobium diazoefficiens* CPAC 7: elite model strains for understanding symbiotic performance with soybean. BMC Genomics.

[7] Dénarié J, Debellé F, Promé J-C (1996). *Rhizobium* lipo-chitooligosaccharide nodulation factors: signaling molecules mediating recognition and morphogenesis. Annu Rev Biochem.

[8] Hungria M, Stacey G (1997). Molecular signals exchanged between host plants and rhizobia: basic aspects and potential application in agriculture. Soil Biol Biochem.

[9] Downie JA, Walker SA (1999). Plant responses to nodulation factors. Curr Opin Plant Biol.

[10] Buhian WP, Bensmihen S, Mini-Review (2018). Nod factor regulation of phytohormone signaling and homeostasis during rhizobia-legume symbiosis. Front Plant Sci.

[11] Pérez-Montaño F, Guasch-Vidal B, González-Barroso S, López-Baena FJ, Cubo T, Ollero FJ (2011). Nodulation-gene-inducing flavonoids increase overall production of autoinducers and expression of N-acyl homoserine lactone synthesis genes in rhizobia. Res Microbiol.

[12] López-Baena FJ, Vinardell JM, Pérez-Montaño F, Crespo-Rivas JC, Bellogín RA, Espuny M del R, et al. Regulation and symbiotic significance of nodulation outer proteins secretion in *Sinorhizobium fredii* HH103. Microbiology. 2008;154:1825–36. 10.1099/mic.0.2007/016337-0. 10.1099/mic.0.2007/016337-0 18524937

[13] Moscatiello R, Squartini A, Mariani P, Navazio L. Flavonoid-induced calcium signalling in *Rhizobium leguminosarum* bv. viciae. New Phytol. 2010;188:814–23. 10.1111/j.1469-8137.2010.03411.x. 10.1111/j.1469-8137.2010.03411.x 20738787

[14] Brencic A, Winans SC (2005). Detection of and response to signals involved in host-microbe interactions by plant-associated bacteria. Microbiol Mol Biol Rev.

[15] Bogino PC, Nievas FL, Giordano W (2015). A review: Quorum sensing in *Bradyrhizobium*. Appl Soil Ecol.

[16] Liu Y, Jiang X, Guan D, Zhou W, Ma M, Zhao B (2017). Transcriptional analysis of genes involved in competitive nodulation in *Bradyrhizobium diazoefficiens* at the presence of soybean root exudates. Sci Rep.

[17] Riviezzi B, Cagide C, Pereira A, Herrmann C, Lombide R, Lage M (2020). Improved nodulation and seed yield of soybean (*Glycine max*) with a new isoflavone-based inoculant of *Bradyrhizobium elkanii*. Rhizosphere.

[18] Ormeño-Orrillo E, Martínez-Romero E (2019). A Genomotaxonomy view of the *Bradyrhizobium* genus. Front Microbiol.

[19] de Lajudie P, Young JPW. International committee on systematics of prokaryotes subcommittee on the taxonomy of rhizobia and agrobacteria minutes of the closed meeting by videoconference, 17 July 2019. Int J Syst Evol Microbiol. 2020;70:3563–71. 10.1099/ijsem.0.004157. 10.1099/ijsem.0.004157 32375927

[20] Pessi G, Ahrens CH, Rehrauer H, Lindemann A, Hauser F, Fischer H-M (2007). Genome-wide transcript analysis of *Bradyrhizobium japonicum* bacteroids in soybean root nodules. Mol Plant Microbe Interact.

[21] Delmotte N, Ahrens CH, Knief C, Qeli E, Koch M, Fischer H-M (2010). An integrated proteomics and transcriptomics reference data set provides new insights into the *Bradyrhizobium japonicum* bacteroid metabolism in soybean root nodules. Proteomics.

[22] Davis-Richardson AG, Russell JT, Dias R, McKinlay AJ, Canepa R, Fagen JR (2016). Integrating DNA methylation and gene expression data in the development of the soybean-*Bradyrhizobium* N_2_-fixing symbiosis. Front Microbiol..

[23] Cytryn EJ, Sangurdekar DP, Streeter JG, Franck WL, Chang W, Stacey G (2007). Transcriptional and physiological responses of *Bradyrhizobium japonicum* to desiccation-induced stress. J Bacteriol.

[24] Wei M, Yokoyama T, Minamisawa K, Mitsui H, Itakura M, Kaneko T (2008). Soybean seed extracts preferentially express genomic loci of *Bradyrhizobium japonicum* in the initial interaction with soybean, *Glycine max* (L.) Merr. DNA Res..

[25] Lang K, Lindemann A, Hauser F, Göttfert M (2008). The genistein stimulon of *Bradyrhizobium japonicum*. Mol Genet Genomics.

[26] Lee H-I, Lee J-H, Park K-H, Sangurdekar D, Chang W-S (2012). Effect of soybean coumestrol on *Bradyrhizobium japonicum* nodulation ability, biofilm formation, and transcriptional profile. Appl Environ Microbiol.

[27] Ohkama-Ohtsu N, Ichida S, Yamaya H, Ohwada T, Itakura M, Hara Y (2015). Peribacteroid solution of soybean root nodules partly induces genomic loci for differentiation into bacteroids of free-living *Bradyrhizobium japonicum* cells. Soil Sci Plant Nutr.

[28] Takeshima K, Hidaka T, Wei M, Yokoyama T, Minamisawa K, Mitsui H (2013). Involvement of a novel genistein-inducible multidrug efflux pump of *Bradyrhizobium japonicum* early in the interaction with *Glycine max* (L.) Merr. Microbes Environ.

[29] Han F, He X, Chen W, Gai H, Bai X, He Y (2020). Involvement of a novel TetR-like regulator (BdtR) of *Bradyrhizobium diazoefficiens* in the efflux of isoflavonoid genistein. Mol Plant-Microbe Interact.

[30] Gomes DF, Batista JS, Rolla AA, da Silva LP, Bloch C, Galli-Terasawa LV (2014). Proteomic analysis of free-living *Bradyrhizobium diazoefficiens*: highlighting potential determinants of a successful symbiosis. BMC Genomics.

[31] Bortolan S, Barcellos FG, Marcelino FC, Hungria M (2009). Expressão dos genes *nodC*, *nodW* e *nop*P em *Bradyrhizobium japonicum* estirpe CPAC 15 avaliada por RT-qPCR. Pesq Agropec Bras.

[32] Guo Gao T, Yuan Xu Y, Jiang F, Zhen Li B, Shui Yang J, Tao Wang E (2015). Nodulation characterization and proteomic profiling of *Bradyrhizobium liaoningense* CCBAU05525 in response to water-soluble humic materials. Sci Rep.

[33] Ratu STN, Teulet A, Miwa H, Masuda S, Nguyen HP, Yasuda M (2021). Rhizobia use a pathogenic-like effector to hijack leguminous nodulation signalling. Sci Rep.

[34] Sen T, Verma NK (2020). Functional annotation and curation of hypothetical proteins present in a newly emerged serotype 1c of *Shigella flexneri*: emphasis on selecting targets for virulence and vaccine design studies. Genes (Basel).

[35] Gomes D, Batista JS da S, Rolla AA, da Silva L, Bloch C, Galli-Terasawa L, et al. Proteomic analysis of free-living *Bradyrhizobium diazoefficiens*: highlighting potential determinants of a successful symbiosis. BMC Genomics. 2014;15:643. 10.1186/1471-2164-15-643. 10.1186/1471-2164-15-643 PMC4287336 25086822

[36] Fong JH, Marchler-Bauer A (2008). Protein subfamily assignment using the conserved domain database. BMC Res Notes.

[37] Ruíz-Valdiviezo VM, Canseco LMCV, Suárez LAC, Gutiérrez-Miceli FA, Dendooven L, Rincón-Rosales R (2015). Symbiotic potential and survival of native rhizobia kept on different carriers. Braz J Microbiol.

[38] Gully D, Gargani D, Bonaldi K, Grangeteau C, Chaintreuil C, Fardoux J (2016). A peptidoglycan-remodeling enzyme is critical for bacteroid differentiation in *Bradyrhizobium* spp. during legume symbiosis. Mol Plant-Microbe Interact.

[39] Vercruysse M, Fauvart M, Jans A, Beullens S, Braeken K, Cloots L (2011). Stress response regulators identified through genome-wide transcriptome analysis of the (p)ppGpp-dependent response in *Rhizobium etli*. Genome Biol.

[40] Quelas JI, Lastra RA, Lorenze C, Escobar M, Lepek VC. Site-directed mutagenesis of *Bradyrhizobium diazoefficiens* USDA 110 *aroA* improves bacterial growth and competitiveness for soybean nodulation in the presence of glyphosate. Environ Microbiol Rep. 2020;1758–2229.12917. 10.1111/1758-2229.12917. 10.1111/1758-2229.12917 33331105

[41] Tullio LD, Gomes DF, Silva LP, Hungria M, Batista JS da S. Proteomic analysis of *Rhizobium freirei* PRF 81^T^ reveals the key role of central metabolic pathways in acid tolerance. Appl Soil Ecol. 2019;135:98–103. 10.1016/j.apsoil.2018.11.014.

[42] Porto de Souza Vandenberghe L, Karp SG, Binder Pagnoncelli MG, von Linsingen Tavares M, Libardi Junior N, Valladares Diestra K, et al. Classification of enzymes and catalytic properties. In: Biomass, Biofuels, Biochemicals. Elsevier; 2020. p. 11–30. 10.1016/B978-0-12-819820-9.00002-8.

[43] Alaminos M, Ramos J. The methionine biosynthetic pathway from homoserine in *Pseudomonas putida* involves the *metW*, *metX*, *metZ*, *metH* and *metE* gene products. Arch Microbiol. 2001;176:151–4. 10.1007/s002030100293. 10.1007/s002030100293 11479715

[44] Yang Q, Li Y, Yang H, Rang J, Tang S, He L (2015). Proteomic insights into metabolic adaptation to deletion of metE in *Saccharopolyspora spinosa*. Appl Microbiol Biotechnol.

[45] Plener L, Boistard P, González A, Boucher C, Genin S (2012). Metabolic adaptation of *Ralstonia solanacearum* during plant infection: a methionine biosynthesis case study. PLoS One.

[46] Mordukhova EA, Pan J-G (2013). Evolved cobalamin-independent methionine synthase (MetE) improves the acetate and thermal tolerance of *Escherichia coli*. Appl Environ Microbiol.

[47] Irazusta V, Estévez C, Amoroso MJ, de Figueroa LIC (2012). Proteomic study of the yeast *Rhodotorula mucilaginosa* RCL-11 under copper stress. BioMetals.

[48] Taga ME, Walker GC (2010). *Sinorhizobium meliloti* requires a cobalamin-dependent ribonucleotide reductase for symbiosis with its plant host. Mol Plant-Microbe Interact.

[49] Percudani R, Peracchi A (2003). A genomic overview of pyridoxal-phosphate‐dependent enzymes. EMBO Rep.

[50] Rastogi VK, Watson RJ (1991). Aspartate aminotransferase activity is required for aspartate catabolism and symbiotic nitrogen fixation in *Rhizobium meliloti*. J Bacteriol.

[51] Watson RJ, Rastogi VK (1993). Cloning and nucleotide sequencing of *Rhizobium meliloti* aminotransferase genes: an aspartate aminotransferase required for symbiotic nitrogen fixation is atypical. J Bacteriol.

[52] Roux B, Rodde N, Jardinaud M-F, Timmers T, Sauviac L, Cottret L (2014). An integrated analysis of plant and bacterial gene expression in symbiotic root nodules using laser-capture microdissection coupled to RNA sequencing. Plant J.

[53] Flores-Tinoco CE, Tschan F, Fuhrer T, Margot C, Sauer U, Christen M (2020). Co‐catabolism of arginine and succinate drives symbiotic nitrogen fixation. Mol Syst Biol.

[54] Da Silva Batista JS, Hungria M (2012). Proteomics reveals differential expression of proteins related to a variety of metabolic pathways by genistein-induced *Bradyrhizobium japonicum* strains. J Proteomics.

[55] Hoshino T, Nakano S, Kondo T, Sato T, Miyoshi A (2004). Squalene–hopene cyclase: final deprotonation reaction, conformational analysis for the cyclization of (3R,S)-2,3-oxidosqualene and further evidence for the requirement of an isopropylidene moiety both for initiation of the polycyclization cascade and for the formation of the 5-membered E-ring. Org Biomol Chem.

[56] Belin BJ, Busset N, Giraud E, Molinaro A, Silipo A, Newman DiK (2018). Hopanoid lipids: from membranes to plant–bacteria interactions. Nat Rev Microbiol.

[57] Kannenberg L (1995). The occurrence of hopanoid lipids in *Bradyrhizobium* bacteria. FEMS Microbiol Lett.

[58] Rosa-Putra S, Nalin R, Domenach A-M, Rohmer M (2001). Novel hopanoids from *Frankia* spp. and related soil bacteria. Eur J Biochem.

[59] Silipo A, Vitiello G, Gully D, Sturiale L, Chaintreuil C, Fardoux J (2014). Covalently linked hopanoid-lipid A improves outer-membrane resistance of a *Bradyrhizobium* symbiont of legumes. Nat Commun.

[60] Lukose V, Walvoort MTC, Imperiali B (2017). Bacterial phosphoglycosyl transferases: initiators of glycan biosynthesis at the membrane interface. Glycobiology.

[61] Lewandowski T, Huang J, Fan F, Rogers S, Gentry D, Holland R (2013). *Staphylococcus aureus* formyl-methionyl transferase mutants demonstrate reduced virulence factor production and pathogenicity. Antimicrob Agents Chemother.

[62] Paul PEV, Sangeetha V, Deepika RG. Emerging trends in the industrial production of chemical products by microorganisms. In: Recent Developments in Applied Microbiology and Biochemistry. Elsevier; 2019. p. 107–25. 10.1016/B978-0-12-816328-3.00009-X.

[63] Schaller M, Borelli C, Korting HC, Hube B (2005). Hydrolytic enzymes as virulence factors of *Candida albicans*. Mycoses.

[64] Dubovenko AG, Dunaevsky YE, Belozersky MA, Oppert B, Lord JC, Elpidina EN (2010). Trypsin-like proteins of the fungi as possible markers of pathogenicity. Fungal Biol.

[65] Prasanth CN, Viswanathan R, Malathi P, Sundar AR (2019). Comparative transcriptome analysis of candidate secretory effector proteins from *Colletotrichum falcatum* infecting sugarcane. Agri Gene.

[66] STORK I, GARTEMANN K-H BURGER A, EICHENLAUB R (2008). A family of serine proteases of *Clavibacter michiganensis* subsp. *michiganensis*: *chpC* plays a role in colonization of the host plant tomato. Mol Plant Pathol.

[67] Redman RS, Rodriguez RJ (2002). Characterization and isolation of an extracellular serine protease from the tomato pathogen *Colletotrichum coccodes*, and it’s role in pathogenicity. Mycol Res.

[68] Rosander A, Frykberg L, Ausmees N, Müller P (2003). Identification of extracytoplasmic proteins in *Bradyrhizobium japonicum* using phage display. Mol Plant-Microbe Interact.

[69] Sarma AD, Emerich DW (2005). Global protein expression pattern of *Bradyrhizobium japonicum* bacteroids: A prelude to functional proteomics. Proteomics.

[70] Meneses N, Mendoza-Hernández G, Encarnación S (2010). The extracellular proteome of *Rhizobium etli* CE3 in exponential and stationary growth phase. Proteome Sci.

[71] Nilsson JF, Castellani LG, Draghi WO, Pérez-Giménez J, Torres Tejerizo GA, Pistorio M (2019). Proteomic analysis of *Rhizobium favelukesii* LPU83 in response to acid stress. J Proteome Res.

[72] Igiehon NO, Babalola OO, Aremu BR (2019). Genomic insights into plant growth promoting rhizobia capable of enhancing soybean germination under drought stress. BMC Microbiol.

[73] Gilbert KB, Vanderlinde EM, Yost CK (2007). Mutagenesis of the carboxy terminal protease CtpA decreases desiccation tolerance in *Rhizobium leguminosarum*. FEMS Microbiol Lett.

[74] Price PA, Tanner HR, Dillon BA, Shabab M, Walker GC, Griffitts JS (2015). Rhizobial peptidase HrrP cleaves host-encoded signaling peptides and mediates symbiotic compatibility. Proc Natl Acad Sci.

[75] Prathiviraj R, Chellapandi P (2020). Evolutionary genetic analysis of unassigned peptidase clan-associated microbial virulence and pathogenesis. Biologia (Bratisl).

[76] Sudtachat N, Ito N, Itakura M, Masuda S, Eda S, Mitsui H (2009). Aerobic vanillate degradation and c1 compound metabolism in *Bradyrhizobium japonicum*. Appl Environ Microbiol.

[77] Ito N, Itakura M, Eda S, Saeki K, Oomori H, Yokoyama T (2006). Global gene expression in *Bradyrhizobium japonicum* cultured with vanillin, vanillate, 4-hydroxybenzoate and protocatechuate. Microbes Environ.

[78] Laranjo M, Alexandre A, Oliveira S (2017). Global transcriptional response to salt shock of the plant microsymbiont *Mesorhizobium loti* MAFF303099. Res Microbiol.

[79] Guerrero-Castro J, Lozano L, Sohlenkamp C (2018). Dissecting the acid stress response of *Rhizobium tropici* CIAT 899. Front Microbiol.

[80] Sugawara M, Sadowsky MJ (2013). Influence of elevated atmospheric carbon dioxide on transcriptional responses of *Bradyrhizobium japonicum* in the soybean rhizoplane. Microbes Environ.

[81] Dunn MF (2015). Key roles of microsymbiont amino acid metabolism in rhizobia-legume interactions. Crit Rev Microbiol..

[82] Prell J, Bourdes a., Karunakaran R, Lopez-Gomez M, Poole P (2009). Pathway of -aminobutyrate metabolism in *Rhizobium leguminosarum* 3841 and its role in symbiosis. J Bacteriol.

[83] Tatè R, Ferraioli S, Filosa S, Cermola M, Riccio A, Iaccarino M (2004). Glutamine utilization by *Rhizobium etli*. Mol Plant-Microbe Interact.

[84] Li J, Epa R, Scott NE, Skoneczny D, Sharma M, Snow AJD, et al. A sulfoglycolytic entner-doudoroff pathway in *Rhizobium leguminosarum* bv. *trifolii* SRDI565. Appl Environ Microbiol. 2020;86:1–15. 10.1128/AEM.00750-20. 10.1128/AEM.00750-20 PMC7376563 32444469

[85] Sarma AD, Emerich DW (2006). A comparative proteomic evaluation of culture grown vs nodule isolated *Bradyrhizobium japonicum*. Proteomics.

[86] Maximiano MR, Megías E, Santos IR, Santos LS, Ollero FJ, Megías M (2021). Proteome responses of *Rhizobium tropici* CIAT 899 upon apigenin and salt stress induction. Appl Soil Ecol.

[87] Epstein B, Abou-Shanab RAI, Shamseldin A, Taylor MR, Guhlin J, Burghardt LT, et al. Genome-wide association analyses in the model *Rhizobium Ensifer meliloti*. mSphere. 2018;3:1–15. 10.1128/mSphere.00386-18. 10.1128/mSphere.00386-18 PMC6200981 30355664

[88] Douglas CD, Ngu TT, Kaluarachchi H, Zamble DB (2013). Metal transfer within the *Escherichia coli* HypB–HypA complex of hydrogenase accessory proteins. Biochemistry.

[89] Kaneko T. Complete genomic sequence of nitrogen-fixing symbiotic bacterium *Bradyrhizobium japonicum* USDA110. DNA Res. 2002;9:189–97. 10.1093/dnares/9.6.189. 10.1093/dnares/9.6.189 12597275

[90] Ormeño-Orrillo E, Menna P, Almeida LGP, Ollero FJ, Nicolás MF, Pains Rodrigues E (2012). Genomic basis of broad host range and environmental adaptability of *Rhizobium tropici* CIAT 899 and *Rhizobium* sp. PRF 81 which are used in inoculants for common bean (*Phaseolus vulgaris* L.). BMC Genomics.

[91] Annan H, Golding A-L, Zhao Y, Dong Z (2012). Choice of hydrogen uptake (Hup) status in legume-rhizobia symbioses. Ecol Evol.

[92] Boddey LH, Hungria M (1997). Phenotypic grouping of Brazilian *Bradyrhizobium* strains which nodulate soybean. Biol Fertil Soils.

[93] Hernando Y, Palacios J, Imperial J, Ruiz-Argueso T (1998). *Rhizobium leguminosarum* bv. *viciae* hypA gene is specifically expressed in pea (*Pisum sativum*) bacteroids and required for hydrogenase activity and processing. FEMS Microbiol Lett.

[94] Franck WL, Chang W, Qiu J, Sugawara M, Sadowsky MJ, Smith SA (2008). Whole-genome transcriptional profiling of *Bradyrhizobium japonicum* during chemoautotrophic growth. J Bacteriol.

[95] Reader JS, Metzgar D, Schimmel P, de Crécy-Lagard V (2004). Identification of four genes necessary for biosynthesis of the modified nucleoside queuosine. J Biol Chem.

[96] Marchetti M, Capela D, Poincloux R, Benmeradi N, Auriac M-C, Le Ru A (2013). Queuosine biosynthesis is required for *Sinorhizobium meliloti*-induced cytoskeletal modifications on hela cells and symbiosis with *Medicago truncatula*. PLoS One.

[97] Durand JMB, Dagberg B, Uhlin BE, Bjork GR (2000). Transfer RNA modification, temperature and DNA superhelicity have a common target in the regulatory network of the virulence of *Shigella flexneri*: the expression of the *virF* gene. Mol Microbiol..

[98] Masi M, Winterhalter M, Pagès J-M. Outer membrane porins. In: Subcellular Biochemistry. Springer International Publishing; 2019. p. 79–123. 10.1007/978-3-030-18768-2_4. 10.1007/978-3-030-18768-2_4 31214985

[99] Saier MH. Cell membrane, Prokaryotic. In: Encyclopedia of Microbiology. Elsevier; 2009. p. 341–56. 10.1016/B978-012373944-5.00049-3.

[100] de María N, Guevara A, Serra MT, García-Luque I, González-Sama A, de Lacoba MG (2007). Putative porin of *Bradyrhizobium* sp. (*Lupinus*) bacteroids induced by glyphosate. Appl Environ Microbiol.

[101] Hohle TH, Franck WL, Stacey G, O’Brian MR (2011). Bacterial outer membrane channel for divalent metal ion acquisition. Proc Natl Acad Sci.

[102] González-Sánchez A, Cubillas CA, Miranda F, Dávalos A, García-de los Santos A (2018). The *ropAe* gene encodes a porin-like protein involved in copper transit in *Rhizobium etli* CFN42. Microbiologyopen.

[103] Abreu I, Mihelj P, Raimunda D (2019). Transition metal transporters in rhizobia: tuning the inorganic micronutrient requirements to different living styles. Metallomics.

[104] Naamala J, Jaiswal SK, Dakora FD (2016). Antibiotics resistance in *Rhizobium*: type, process, mechanism and benefit for agriculture. Curr Microbiol..

[105] Cabrera JJ, Salas A, Torres MJ, Bedmar EJ, Richardson DJ, Gates AJ (2016). An integrated biochemical system for nitrate assimilation and nitric oxide detoxification in *Bradyrhizobium japonicum*. Biochem J..

[106] Musa MA, Wahab RA, Huyop F (2018). Homology modelling and *in silico* substrate-binding analysis of a *Rhizobium* sp. RC1 haloalkanoic acid permease. Biotechnol Biotechnol Equip.

[107] Eda S, Mitsui H, Minamisawa K (2011). Involvement of the SmeAB multidrug efflux pump in resistance to plant antimicrobials and contribution to nodulation competitiveness in *Sinorhizobium meliloti*. Appl Environ Microbiol.

[108] Frederix M, Edwards A, Swiderska A, Stanger A, Karunakaran R, Williams A (2014). Mutation of in *Rhizobium leguminosarum* enhances root biofilms, improving nodulation competitiveness by increased expression of attachment proteins. Mol Microbiol..

[109] Bartlett EJ, Doherty AJ. Nonhomologous end joining in bacteria. In: Lennarz WJ, Lane MDBT-E of BC (Second E, editors. Encyclopedia of Biological Chemistry. Waltham: Elsevier; 2013. p. 266–8. 10.1016/B978-0-12-378630-2.00407-2.

[110] Kobayashi H, Simmons LA, Yuan DS, Broughton WJ, Walker GC (2007). Multiple Ku orthologues mediate DNA non-homologous end-joining in the free-living form and during chronic infection of *Sinorhizobium meliloti*. Mol Microbiol.

[111] Dupuy P, Sauviac L, Bruand C (2019). Stress-inducible NHEJ in bacteria: function in DNA repair and acquisition of heterologous DNA. Nucleic Acids Res.

[112] Dupuy P, Gourion B, Sauviac L, Bruand C (2017). DNA double-strand break repair is involved in desiccation resistance of *Sinorhizobium meliloti*, but is not essential for its symbiotic interaction with *Medicago truncatula*. Microbiology.

[113] Yurimoto H, Hirai R, Matsuno N, Yasueda H, Kato N, Sakai Y (2005). HxlR, a member of the DUF24 protein family, is a DNA-binding protein that acts as a positive regulator of the formaldehyde-inducible *hxlAB* operon in *Bacillus subtilis*. Mol Microbiol.

[114] Gómez-Sagasti MT, Becerril JM, Epelde L, Alkorta I, Garbisu C (2015). Early gene expression in *Pseudomonas fluorescens* exposed to a polymetallic solution. Cell Biol Toxicol.

[115] Yang X, Teng K, Zhang J, Wang F, Zhang T, Ai G (2017). Transcriptome responses of *Lactobacillus acetotolerans* F28 to a short and long term ethanol stress. Sci Rep.

[116] Mwita L, Chan WY, Pretorius T, Lyantagaye SL, Lapa SV, Avdeeva LV (2016). Gene expression regulation in the plant growth promoting *Bacillus atrophaeus* UCMB-5137 stimulated by maize root exudates. Gene.

[117] Uchiumi T, Ohwada T, Itakura M, Mitsui H, Nukui N, Dawadi P (2004). Expression islands clustered on the symbiosis island of the *Mesorhizobium loti* Genome. J Bacteriol.

[118] Kaneko T, Maita H, Hirakawa H, Uchiike N, Minamisawa K, Watanabe A (2011). Complete genome sequence of the soybean symbiont *Bradyrhizobium japonicum* strain USDA6^T^. Genes (Basel).

[119] Ormeño-Orrillo E, Martínez-Romero E, Zúñiga-Dávila D (2020). Identification of the symbiosis island of *Bradyrhizobium paxllaeri* LMTR 21^T^. Braz J Microbiol.

[120] Roche P, Maillet F, Plazanet C, Debelle F, Ferro M, Truchet G (1996). The common *nodABC* genes of *Rhizobium meliloti* are host-range determinants. Proc Natl Acad Sci USA.

[121] Loh J, Stacey G (2003). Nodulation gene regulation in *Bradyrhizobium japonicum*: a unique integration of global regulatory circuits. Appl Environ Microbiol.

[122] Cooper JE (2007). Early interactions between legumes and rhizobia: disclosing complexity in a molecular dialogue. J Appl Microbiol..

[123] Kobayashi H, Graven YN, Broughton WJ, Perret X (2004). Flavonoids induce temporal shifts in gene-expression of nod-box controlled loci in *Rhizobium* sp. NGR234. Mol Microbiol.

[124] Kullik I, Fritsche S, Knobel H, Sanjuan J, Hennecke H (1991). *Bradyrhizobium japonicum* has two differentially regulated, functional homologs of the sigma-54 gene (RpoN). J Bacteriol.

[125] Hauser F, Pessi G, Friberg M, Weber C, Rusca N (2007). Dissection of the *Bradyrhizobium japonicum* NifA + sigma54 regulon, and identification of a ferredoxin gene (*fdxN*) for symbiotic nitrogen fixation. Mol Genet Genomics.

[126] Čuklina, J., Hahn, J., Imakaev, M., et al. Genome-wide transcription start site mapping of *Bradyrhizobium japonicum* grown free-living or in symbiosis – a rich resource to identify new transcripts, proteins and to study gene regulation. BMC Genomics. 2016;17:302 (2016). 10.1186/s12864-016-2602-9. 10.1186/s12864-016-2602-9 PMC4842269 27107716

[127] Beck C, Marty R, Kläusli S, Hennecke H, Göttfert M (1997). Dissection of the transcription machinery for housekeeping genes of *Bradyrhizobium japonicum*. J Bacteriol.

[128] Meier D, Casas-Pastor D, Fritz G, Becker A (2020). Gene regulation by extracytoplasmic function (ECF) o factors in alpha-rhizobia. Adv Bot Res.

[129] Clark SRD, Oresnik IJ, Hynes MF RpoN of *Rhizobium leguminosarum* bv. *viciae* strain VF39SM plays a central role in FnrN-dependent microaerobic regulation of genes involved in nitrogen fixation. Mol Gen Genet. 2001;264:623–33. 10.1007/s004380000348. 10.1007/s004380000348 11212917

[130] Dombrecht B, Marchal K, Vanderleyden J, Michiels J (2002) Prediction and overview of the RpoN-regulon in closely related species of the Rhizobiales. Genome Biol. 2002;3:0076.0071. 10.1186/gb-2002-3-12-research0076. 10.1186/gb-2002-3-12-research0076 PMC151178 12537565

[131] Tully RE, van Berkum P, Lovins KW, Keister DL. Identification and sequencing of a cytochrome P450 gene cluster from *Bradyrhizobium japonicum.* Biochim Biophys Acta – Gene Struct Expre. 1998;1398:243–55. 10.1016/s0167-4781(98)00069-4. 10.1016/s0167-4781(98)00069-4 9655913

[132] Sullivan JT, Brown SD, Ronson CW (2013). The NifA-RpoN regulon of *Mesorhizobium loti* strain R7A and its symbiotic activation by a novel LacI/GalR-family regulator. PLoS One.

[133] O’Leary NA, Wright MW, Brister JR, Ciufo S, Haddad D, McVeigh R (2016). Reference sequence (RefSeq) database at NCBI: current status, taxonomic expansion, and functional annotation. Nucleic Acids Res.

[134] Tamura K, Stecher G, Kumar S (2021). MEGA11: Molecular evolutionary genetics analysis version 11. Mol Biol Evol.

[135] Saitou N, Nei M (1987). The neighbor-joining method: a new method for reconstructing phylogenetic trees. Mol Biol Evol.

[136] Wang JY, Wang R, Zhang YM, Liu HC, Chen WF, Wang ET (2013). *Bradyrhizobium daqingense* sp. nov., isolated from soybean nodules. Int J Syst Evol Microbiol.

[137] Hungria M, O’Hara G, Zilli J, Araujo RS, Deaker R, Howieson J (2016). Isolation and growth or rhizobia. Working with rhizobia.

[138] Beringer JE (1974). R Factor transfer in *Rhizobium leguminosarum*. Microbiology.

[139] Broeders S, Huber I, Grohmann L, Berben G, Taverniers I, Mazzara M (2014). Guidelines for validation of qualitative real-time PCR methods. Trends Food Sci Technol.

[140] Pfaffl MW (2002). Relative expression software tool (REST(C)) for group-wise comparison and statistical analysis of relative expression results in real-time PCR. Nucleic Acids Res.

[141] Gomes DF, Batista JSDS, Schiavon AL, Andrade DS, Hungria M (2012). Proteomic profiling of *Rhizobium tropici* PRF 81: identification of conserved and specific responses to heat stress. BMC Microbiol.

