# Supplementary material for: Revealing potential functions of hypothetical proteins induced by genistein in the symbiosis island of Bradyrhizobium japonicum commercial strain SEMIA 5079 (= CPAC 15)
Source: BMC Microbiol. 2022 May 5;22:122. doi: 10.1186/s12866-022-02527-9 (PMC9069715; doi:10.1186/s12866-022-02527-9)
Supplement: Supplementary file 2 — Additional file 2. [file 12866_2022_2527_MOESM2_ESM.docx]

**Supplementary file 2**. Inference functional of the HPs chosen in the present study. All the information collected for each protein has been shown. In parentheses was described the databases and protein domain associated with each HP.

| **Hypothetical Protein** | **GenBank** | **Predicted function** |
| --- | --- | --- |
|  |  |  |
| **BJS_07647** | AHY48641.1 |  |
| **Protein**: Porin protein  **Pfam:** Outer membrane protein beta-barrel domain, OMP_b-brl (PF13505)  **SUPFAM:** OMPA-like superfamily (SSF56925)  **InterPro:** OMP/PagP_b-brl (IPR011250); OMP_b-brl (IPR027385)  **General Category:** Processes_IC (SUPFAM)  **Detailed Category:** Transport (SUPFAM)  **Description:** Porin superfamily. These outer membrane channels share a beta-barrel structure that differ in strand and shear number. Classical (gram-negative) porins are non-specific channels for small hydrophilic molecules and form 16 beta-stranded barrels (16,20). Porin family protein is an outer-membrane immunogenic protein similar to opacity protein LomR and related surface antigens (NCBI).  **Biological process** – molecules transport (HGNC); Gram-negative-bacterium-type cell outer membrane assembly (GO:0043165)  **Molecular function:** porins are non-specific channels for small hydrophilic molecules and form 16 beta-stranded barrels (16,20), which associate as trimers. (NCBI) | | |
| **Protein** | **GenBank** | **Predicted function** |
| **BJS_07621** | AHY48670.1 |  |
| **Protein**: Glutathione-dependent formaldehyde-activating (GFA) enzyme  **Pfam:** GFA (PF04828)  **SUPFAM:** Mss4-like superfamily (SSF51316)  **InterPro**: CENP-V/GFA (IPR006913); Haem_Oase-like_multi-hlx (IPR016084); Mss4-like_sf (IPR011057)  **PROSITE**: CENP_V_GFA (PS51891)  **General Category:** Regulation (SUPFAM)  **Detailed Category:** Signal transduction (SUPFAM)  **Description**: Glutathione-dependent formaldehyde-activating enzyme GFA catalyzes the condensation of formaldehyde and glutathione to S-hydroxymethylglutathione (InterPro).  **Molecular function:** carbon – sulfur lyase activity (GO:0016846).  **Biological process:** metabolic process (GO), formaldehyde-detoxification pathway.  **KEGG**: EC 4.4.1.22 | | |
| **Protein** | **GenBank** | **Predicted function** |
| **BJS_07605** | AHY48686.1 |  |
| **Protein**: 5-methyltetrahydropteroyltriglutamate--homocysteine methyltransferase  **Gene**: *metE*  **Pfam:** Cobalamin-independent synthase, N-terminal domain, Meth_synt_1 (PF08267); Meth_synt_2, cobalamin-independent synthase, catalytic domain (PF01717)  **SUPFAM**: UROD/MetE-like superfamily (SSF51726)  **InterPro**: Cbl-indep_Met_Synth_N (IPR013215); Cobalamin-indep_Met_synthase (IPR006276); Met_Synth_C/arc (IPR002629); UROD/MetE-like_sf (IPR038071)  **General Category:** Metabolism (SUPFAM)  **Detailed Category:** Coenzyme metabolism and transport (SUPFAM)  **Description**: This enzyme catalyses the last step in the production of methionine by transferring a methyl group from 5-methyltetrahydrofolate to L-homocysteine without using an intermediate methyl carrier (NCBI).  **Molecular function:** 5-methyltetrahydropteroyltriglutamate-homocysteine S-methyltransferase activity (GO:0003871); Methyltransferase; zinc ion binding (GO:0008270)  **Biological Process**: Methionine biosynthesis process (GO:0009086), methylation (GO:0032259)  **EC number:** EC 2.1.1.14 (KEGG) | | |
| **Protein** | **GenBank** | **Predicted function** |
| **BJS_07536** | AHY48760.1 |  |
| **Protein:** Squalene-hopene cyclase  **Pfam**: SQHop_cyclase_C (PF13243)  **SUPFAM**: Terpenoid cyclases/Protein prenyltransferases superfamily (SSF48239)  **InterPro**: Terpenoid_cyclase/PrenylTrfase (IPR008930)  **General Category:** Metabolism (SUPFAM)  **Detailed Category:** Secondary Metabolism (SUPFAM)  **Description**: It has been suggested that this gene family be designated tps (for terpene synthase). It has been split into six subgroups on the basis of phylogeny, called tpsa-tpsf. tpsa includes vetispiridiene synthase, 5-epi- aristolochene synthase, and (+)-delta-cadinene synthase. tpsb includes (-)-limonene synthase. tpsc includes kaurene synthase A. tpsd includes taxadiene synthase, pinene synthase, and myrcene synthase. tpse includes kaurene synthase B. tpsf includes linalool synthase (NCBI).  **Molecular function***:* it directly cyclizes squalene into hopanoid products (NCBI).  **Biological process**: Fatty acid and phospholipid metabolism (NCBI); hopanoid (triterpenoid) metabolism (InterPro) (GO:0006722)  **KEGG**: EC 5.4.99.17 | | |
| **Protein** | **GenBank** | **Predicted function** |
| **BJS_08774** | AHY56918.1 |  |
| **Protein**: Acetylcholinesterase  **Pfam**: AChE_tetra, Acetylcholinesterase tetramerization domain (PF08674)  **SUPFAM**: no results  **InterPro**: Acetylcholinesterase (IPR014788)  **Description:** The acetylcholinesterase tetramerization domain is found at the C terminus and forms a left handed superhelix.  **Description**: Acts on a variety of acetic esters; also catalyses transacetylations (KEGG)  **Molecular function**: hydrolases; acting on ester bonds; carboxylic-ester hydrolases (KEGG)  **Biological process**: glycerophospholipid metabolism (KEGG)  **KEGG:** EC 3.1.1.7 | | |
| **Protein** | **GenBank** | **Predicted function** |
| **BJS_08523** | AHY56944.1 |  |
| **Protein**: 7-cyano-7-deazaguanine synthase, also known as queuosine biosynthesis protein  **Pfam**: QueC, Queuosine biosynthesis protein (PF06508); Tricorn_C1, Tricorn protease C1 ()  **InterPro**: Rossmann-like_a/b/a_fold (IPR014729); HUPs (G3DSA:3.40.50.620); Adenine nucleotide alpha hydrolases-like (SSF52402)    **Description**: Queuosine biosynthesis protein (QueC), also known as 7-cyano-7-deazaguanine synthase, catalyzes the ATP-dependent conversion of 7-carboxy-7-deazaguanine (CDG) to 7-cyano-7-deazaguanine (preQ0)  **Molecular function**: catalyzes the ATP-dependent conversion of 7-carboxy-7-deazaguanine (CDG) to 7-cyano-7-deazaguanine (preQ0)  **Biological** **process**: queuosine biosynthetic process (GO:0008616)  **KEGG**: EC 6.3.4.20 | | |
| **Protein** | **GenBank** | **Predicted function** |
| **BJS_08160** | AHY56892.1 |  |
| **Protein**: Non-homologous end joining protein Ku  **Gene**: ku  **Pfam**: Ku70/Ku80 beta-barrel domain (PF02735)  **InterPro**: Ku70/Ku80_beta-barrel_dom (IPR006164)  **SUPERFAM**: SPOC domain-like superfamily (SSF100939)  **General Category:** Regulation (SUPFAM)  **Detailed Category:** Signal transduction (SUPFAM)  **Description**: Protein composed of prokaryotic homologs of the eukaryotic DNA binding protein Ku. The alignment includes the core domain shared by the prokaryotic YkoV-like proteins and the eukaryotic Ku70 and Ku80. The prokaryotic Ku homologs are predicted to form homodimers. It is proposed that the Ku homologs are functionally associated with ATP-dependent DNA ligase and the eukaryotic-type primase, probably as components of a double-strand break repair system (NCBI).  **Molecular** **function**: DNA binding (GO:0003677)  **Biological** **process**: Double-strand break repair via nonhomologous end joining (GO:0006303); DNA recombination (UniProt) | | |
| **Protein** | **GenBank** | **Predicted function** |
| **BJS_08317** | **AHY48455.1** |  |
| **Pfam**: HxlR-like helix-turn-helix (PF01638)  **InterPro**: hxlR-type HTH (IPR002577)  **Description**: The hxlR-type HTH domain is a domain of ~90-100 amino acids present in putative transcription regulators with a winged helix-turn-helix (wHTH) structure. The domain is named after *Bacillus* *subtilis* hxlR, a transcription activator of the hxlAB operon involved in the detoxification of formaldehyde (InterPro)  *Molecular function*: transcription regulator  *Biological process:* amino acid biosynthesis | | |
| **Protein** | **GenBank** | **Predicted function** |
| **BJS_08261** | **AHY48511.1** |  |
| **Protein**: Succinate-semialdehyde dehydrogenase [NADP+]  **Gene**: *gabD*  **Pfam**: Aldedh, Aldehyde dehydrogenase family (PF00171)  **InterPro**: Aldehyde dehydrogenase domain (IPR015590)  **SUPFAM**: ALDH-like superfamily (SSF53720)  **General** **category**: Metabolism (SUPFAM)  **Detailed** **category**: Redox (SUPFAM)  **Description**: Catalysis of the reaction: succinate semialdehyde + NAD(P)+ + H_2_O = succinate + NAD(P)H + H+. (GO)  **Molecular** **function**: succinate-semialdehyde dehydrogenase [NAD(P)+] activity (GO:0009013), Oxidoreductase (Uniprot)  **Biological process**: Oxidation-reduction process (GO:0055114)  **KEGG**: EC 1.2.1.24 | | |
| **Protein** | **GenBank** | **Predicted function** |
| **BJS_08258** | **AHY48514.1** |  |
| **Protein**: Aminotransferase class I/II-fold pyridoxal phosphate-dependent enzyme  **Gene:** *aspC*  **Pfam**: Aminotran_1_2, Aminotransferase class I and II (PF00155)  **InterPro**: Aminotransferase_I/II (IPR004839)  **SUPFAM**: PLP-dependent transferases superfamily (SSF53383)  **General** **category**: Metabolism (SUPFAM)  **Detailed** **category**: Transferases (SUPFAM)  **Description**: Aminotransferases share certain mechanistic features with other pyridoxal-phosphate dependent enzymes, such as the covalent binding of the pyridoxal-phosphate group to a lysine residue. On the basis of sequence similarity, these various enzymes can be grouped into class I and class II. This entry includes proteins from both subfamilies (InterPro).  **Molecular** **function**: pyridoxal phosphate binding (GO:0030170), transaminase activity (GO:0008483).  **Biological** **process**: biosynthetic process (GO:0009058)  **KEGG**: EC 2.6.1.1 | | |
| **Protein** | **GenBank** | **Predicted function** |
| **BJS_08254** | **AHY48517.1** |  |
| **Protein**: Trypsin-like peptidase domain-containing protein  **Pfam**: Trypsin_2, Trypsin-like peptidase domain (PF13365)  **InterPro**: Peptidase_S1B (IPR008256); Trypsin_dom (IPR001254); Serine Protease (PTHR15462)  **Prosite**: V8_HIS, Serine proteases, V8 family, histidine active site (PS00672); TRYPSIN_HIS, Serine proteases, trypsin family, histidine active site (PS00134).  **Description**: Catalysis of the hydrolysis of peptide bonds in a polypeptide chain by a catalytic mechanism that involves a catalytic triad consisting of a serine nucleophile that is activated by a proton relay involving an acidic residue (e.g. aspartate or glutamate) and a basic residue (usually histidine) (GO).  **Molecular** **function**: serine-type peptidase activity (GO:0008236); serine-type endopeptidase activity (GO:0004252)  **Biological** **process**: proteolysis (GO:0006508).  **KEGG**: EC 3.4.21.4 | | |
| **Protein** | **GenBank** | **Predicted function** |
| **BJS_08251** | **AHY48520.1** |  |
| **Protein**: Trypsin-like peptidase domain-containing protein  **Pfam**: Trypsin_2, Trypsin-like peptidase domain (PF13365)  **InterPro**: Peptidase_S1_PA (IPR009003)  **SUPFAM**: Trypsin-like serine proteases superfamily (SSF50494)  **General** **category**: Processes_IC (SUPFAM)  **Detailed** **category**: Proteases (SUPFAM)  **Description**: Serine protease similar to *Escherichia coli* periplasmic serine peptidase DegS, a trypsin-like protease that functions as a trimer (NCBI).  **Molecular** **function**: serine-type endopeptidase activity (GO:0004252).  **Biological** **process**: Hydrolase, Protease (GO:0006508).  **KEGG**: EC 3.4.21.4 | | |
| **Protein** | **GenBank** | **Predicted function** |
| **BJS_08240** | **AHY48531.1** |  |
| **Pfam**: Peptidase U49 (PF10463)  **InterPro**: Peptidase U49, Lit peptidase (IPR019504)  **Description**: This family contains Lit peptidase from Escherichia coli. Lit protease functions in bacterial cell death in response to infection by bacteriophage T4. Following binding of Gol peptide to domains II and III of elongation factor Tu, the Lit peptidase cleaves domain I of the elongation factor. This prevents binding of guanine nucleotides, shuts down translation and leads to cell death (Pfam)  **Molecular function**: Proteolytic function (InterPro).  **Biological** **process**: Catalytic mechanism (Uniprot). | | |
| **Protein** | **GenBank** | **Predicted function** |
| **BJS_08216** | **AHY48554.1** |  |
| **Protein:** Hydrogenase maturation factor HypA  **Pfam**: HypA, Hydrogenase/urease nickel incorporation, metallochaperone (PF01155); Zn-ribbon_8, Zinc ribbon domain (PF09723).  **InterPro**: Hydrogenase maturation factor HypA/HybF (IPR000688); Putative regulatory protein, FmdB, Zinc ribbon domain (IPR013429)  **Description**: Bacterial membrane-bound nickel-dependent hydrogenases require a number of accessory proteins which are involved in their maturation. One of these proteins is generally known as HypA. HypA is a metallochaperone that binds nickel to bring it safely to its target. The nickel coordinates with four nitrogens within the protein. Four conserved cysteines towards the C terminus bind one zinc moiety, probably to stabilise the protein fold  **Molecular function**: nickel cation binding (GO:0016151); zinc ion binding (GO:0008270)  **Biological process:** cellular protein modification process (GO:0006464); protein maturation (GO:0051604) | | |
| **Protein** | **GenBank** | **Predicted function** |
| **BJS_08267** | **AHY48505.1** |  |
| **Protein**: MFS transporter  **Pfam**: MFS_1, Major Facilitator Superfamily (PF07690)  **InterPro**: Major facilitator superfamily (IPR011701)  **SUPFAM**: MFS general substrate transporter superfamily (SSF103473)  **General** **category**: Processes_IC (SUPFAM)  **Detailed** **category**: Ion metabolism and transport (SUPFAM)  **Description**: The Major Facilitator Superfamily (MFS) is a large and diverse group of secondary transporters that includes uniporters, symporters, and antiporters. MFS proteins facilitate the transport across cytoplasmic or internal membranes of a variety of substrates including ions, sugar phosphates, drugs, neurotransmitters, nucleosides, amino acids, and peptides (NCBI).  **Molecular function**: transmembrane transporter activity (GO:0022857)  **Biological process**: transmembrane transport (GO:0055085) | | |
