# Supplementary material for: Revealing potential functions of hypothetical proteins induced by genistein in the symbiosis island of Bradyrhizobium japonicum commercial strain SEMIA 5079 (= CPAC 15)
Source: BMC Microbiol. 2022 May 5;22:122. doi: 10.1186/s12866-022-02527-9 (PMC9069715; doi:10.1186/s12866-022-02527-9)
Supplement: Supplementary file 5 — Additional file 5. [file 12866_2022_2527_MOESM5_ESM.docx]

**Supplementary File 5a** - Phylogenetic tree of the BJS_08317 protein localized in the symbiotic island of *Bradyrhizobium japonicum* strain SEMIA 5079 (=CPAC 15). Only bootstrap values over 70% are shown at the nodes.

**A) BJS_08317**

**Supplementary File 5b** - Phylogenetic tree of the BJS_08261 protein localized in the symbiotic island of *Bradyrhizobium japonicum* strain SEMIA 5079 (=CPAC 15). Only bootstrap values over 70% are shown at the nodes.

# B) BJS_08261

**Supplementary File 5c** - Phylogenetic tree of the BJS_08258 protein localized in the symbiotic island of *Bradyrhizobium japonicum* strain SEMIA 5079 (=CPAC 15). Only bootstrap values over 70% are shown at the nodes.

# C) BJS_08258

**Supplementary File 5d** - Phylogenetic tree of the BJS_08254 protein localized in the symbiotic island of *Bradyrhizobium japonicum* strain SEMIA 5079 (=CPAC 15). Only bootstrap values over 70% are shown at the nodes.

# D) BJS_08254

**Supplementary File 5e** - Phylogenetic tree of the BJS_08251 protein localized in the symbiotic island of *Bradyrhizobium japonicum* strain SEMIA 5079 (=CPAC 15). Only bootstrap values over 70% are shown at the nodes.

# E) BJS_08251

**Supplementary File 5f** - Phylogenetic tree of the BJS_07647 protein localized in the symbiotic island of *Bradyrhizobium japonicum* strain SEMIA 5079 (=CPAC 15). Only bootstrap values over 70% are shown at the nodes.

# F) BJS_07647

**Supplementary File 5g** - Phylogenetic tree of the BJS_07621 protein localized in the symbiotic island of *Bradyrhizobium japonicum* strain SEMIA 5079 (=CPAC 15). Only bootstrap values over 70% are shown at the nodes.

# G) BJS_07621

**Supplementary File 5h** - Phylogenetic tree of the BJS_07605 protein localized in the symbiotic island of *Bradyrhizobium japonicum* strain SEMIA 5079 (=CPAC 15). Only bootstrap values over 70% are shown at the nodes.

# H) BJS_07605

**Supplementary File 5i** - Phylogenetic tree of the BJS_07536 protein localized in the symbiotic island of *Bradyrhizobium japonicum* strain SEMIA 5079 (=CPAC 15). Only bootstrap values over 70% are shown at the nodes.

# I) BJS_07536

**Supplementary File 5j** - Phylogenetic tree of the BJS_08774 protein localized in the symbiotic island of *Bradyrhizobium japonicum* strain SEMIA 5079 (=CPAC 15). Only bootstrap values over 70% are shown at the nodes.

# J) BJS_08774

**Supplementary File 5k** - Phylogenetic tree of the BJS_08523 protein localized in the symbiotic island of *Bradyrhizobium japonicum* strain SEMIA 5079 (=CPAC 15). Only bootstrap values over 70% are shown at the nodes.

# K) BJS_08523

**Supplementary File 5l** - Phylogenetic tree of the BJS_08240 protein localized in the symbiotic island of *Bradyrhizobium japonicum* strain SEMIA 5079 (=CPAC 15). Only bootstrap values over 70% are shown at the nodes.

# L) BJS_08240

**Supplementary File 5m** - Phylogenetic tree of the BJS_08216 protein localized in the symbiotic island of *Bradyrhizobium japonicum* strain SEMIA 5079 (=CPAC 15). Only bootstrap values over 70% are shown at the nodes.

# M) BJS_08216

**Supplementary File 5o** - Phylogenetic tree of the BJS_08267 protein localized in the symbiotic island of *Bradyrhizobium japonicum* strain SEMIA 5079 (=CPAC 15). Only bootstrap values over 70% are shown at the nodes.

# O) BJS_08267

**Supplementary File 5p** - Phylogenetic tree of the BJS_08160 protein localized in the symbiotic island of *Bradyrhizobium japonicum* strain SEMIA 5079 (=CPAC 15). Only bootstrap values over 70% are shown at the nodes.

# P) BJS_8160
